# Rare Disease Drug Repurposing

**DOI:** 10.1001/jamanetworkopen.2025.8330

**Published:** 2025-05-05

**Authors:** Sally Nijim, Ania Korsunska, Joseph Zinski, Sarah E. Bolden, Mary Zuccato, Mileva Repasky, David C. Fajgenbaum

**Affiliations:** 1 Castleman Disease Collaborative Network, Paso Robles, California; 2 Center for Cytokine Storm Treatment and Laboratory, University of Pennsylvania Perelman School of Medicine, Philadelphia; 3 School of Information Studies, Syracuse University, Syracuse, New York

## Abstract

**Importance:**

Treatments are urgently needed for the more than 9500 rare diseases with no US Food and Drug Administration–approved therapies. Although repurposing can be less time- and cost-intensive compared with novel drug development, hurdles have impeded systematic repurposing. Rare disease nonprofit organizations (RDNPs) are well-positioned to overcome barriers and have spearheaded rare disease repurposing efforts for decades. However, no comprehensive data are available on the state of rare disease repurposing or features of successful efforts.

**Objective:**

To characterize the state of rare disease drug repurposing, identify factors associated with successful outcomes, and share thematic insights using the interactive Repurposing of All Drugs, Mapping All Paths (ROADMAP) Project web tool.

**Design, Setting, and Participants:**

The ROADMAP study was a qualitative study using a mixed-methods analysis of US-based RDNP leaders and their stakeholders, including a national survey and semistructured interviews of RDNP leaders, conducted from September 29, 2021, to January 6, 2022. Surveys and interviews revealed themes associated with RDNP strategies, timelines, and support mechanisms. Data were analyzed from January 22, 2024, to April 23, 2024.

**Main Outcomes and Measures:**

The primary survey outcome was the repurposing project stage (abandoned, early, clinical, late, or successful). Qualitative outcomes included themes characterizing repurposing experiences. Two random forest models of drug- and disease- specific as well as organization-specific variables were used to evaluate factor importance toward inferring the project stage. Orthogonal significance testing was conducted using Spearman rank correlation, and *P* values in each model were corrected for multiple hypothesis testing using a Benjamini-Hochberg procedure.

**Results:**

Representative organizations submitted survey responses, including 147 of 698 potential US-based RDNPs. The median RDNP age was 10 years (IQR, 5-20 years), and the median annual revenue was $355 390 (IQR, $90 028-$946 108). Among 34 leaders who were interviewed, representing 25 RDNPs, 23 were female (67.6%), and the RDNPs had a median age of 15 years (IQR, 6-19 years) and a median revenue of $670 719 (IQR, $193 587-$1 830 890). Among the surveyed RDNPs, 58 of 138 (42.0%) specifically identifying their involvement in repurposing supported repurposing projects, and 94 drugs were in various stages of repurposing, of which 23 met success criteria (5 with US Food and Drug Administration approval and 18 with off-label use with subjective benefit). Survey factors associated with successful outcomes included nonprofit-supported patient recruitment into trials (Gini importance, 3.90; ρ = 0.50; adjusted *P* < .001) and provision of nonfinancial research support (Gini importance, 0.69; ρ = 0.33; adjusted *P* = .02). Interview themes were synthesized into a 5-stage repurposing framework with roadblocks and recommendations that included (1) enabling drug repurposing, (2) identifying a drug therapy, (3) validating a drug therapy, (4) clinical use and testing, and (5) reaching an optimal end point for clinical practice.

**Conclusions and Relevance:**

The findings of this qualitative study of RDNP repurposing suggest that several opportunities were associated with successful outcomes and can be considered to optimize systematic repurposing among RDNPs, external collaborators, and policymakers with the use of an interactive tool showcasing insights to facilitate data-driven drug repurposing.

## Key Points

**Question:**

What are common themes among US-based rare disease repurposing efforts, and what factors are associated with successful projects?

**Findings:**

This qualitative study that included a survey of 147 participating rare disease nonprofit organization leaders and 25 semistructured interviews reported on 94 repurposing projects at various stages. Five chronologic themes described stages of repurposing; roadblocks and recommendations were identified from interviews; and key factors, including nonprofit-supported patient recruitment into trials, were associated with successful outcomes.

**Meaning:**

These findings highlight opportunities to optimize systematic repurposing for rare disease nonprofit organizations, external collaborators, and policymakers while providing a framework and tool for data-driven drug repurposing.

## Teaser

This qualitative study, using a mixed-methods analysis that included a survey and interviews, assessed themes among rare disease repurposing efforts in the US and factors associated with successful projects.

## Introduction

Over 10 000 rare diseases affect approximately 30 000 000 individuals in the US.^1,2^ However, 95% of rare diseases do not have a US Food and Drug Administration (FDA)-approved therapy,^3^ highlighting the shortcomings of traditional drug development and the unique challenges that rare disease researchers face.

Drug repurposing, which is the process of identifying new uses for already-approved drugs, is a promising approach for identifying new treatments due to its biologic potential, relatively favorable risk profile, and feasibility.^4,5,6^ The biologic premise underlying drug repurposing is that many diseases share similar or otherwise overlapping mechanisms (eg, idiopathic multicentric Castleman disease and rheumatoid arthritis, both of which involve excess interleukin-6 signaling), and therefore treatment with the same drug is possible (eg, tocilizumab, an interleukin-6 inhibitor).^7,8,9^ Furthermore, many drugs modulate multiple targets (eg, thalidomide depletes Ikaros and casein kinase 1α), each with potential roles across multiple diseases.^10,11^ Repurposing is attractive, as it fundamentally uses known drugs with well-characterized efficacy and safety profiles. This reduces many early-phase uncertainties of novel drug testing and has the potential to mitigate the high attrition rates, cost, and slow pace of new drug development.^12,13^ Physicians often prescribe drugs off-label based on hypothesized mechanisms, anecdotal clinical data, and/or treatment guidelines, which further facilitates repurposing.^14,15,16^ In the US, 20% to 32% of prescriptions are off-label and can constitute up to 80% of prescriptions in populations like neonates.^16,17^

While repurposing is a promising strategy for advancing treatments, it is particularly attractive for rare diseases, which have a disproportionate therapeutic need and reduced investment allocated toward novel drug discovery.^18^ Despite its potential, repurposing is underused. Several barriers prevent repurposing from occurring more frequently and systematically, including siloed data containing potential drug–disease links and off-label drug use; insufficient financial incentives, business models, and/or funding for repurposing, particularly for the more than 80% of drugs that are generic; and lack of an entity responsible for ensuring that drugs are repurposed for all diseases that they can treat.^19,20,21^ There is also confusion regarding repurposing and potential paths. There is no unifying definition for drug repurposing, and 1 review found more than 10 definitions varying in scope, with no consistent unifying definition or approach applied.^22^ For rare diseases, additional challenges include patient sparsity, disease heterogeneity, and limited pathophysiologic understanding.^23^

Rare disease nonprofit organizations (RDNPs) are well-positioned to address these barriers and, in collaboration with academic researchers and companies, have spearheaded rare disease repurposing for decades.^24,25,26^ However, these projects vary in approaches, resources, and outcomes, and there are limited data available to characterize the state of rare disease drug repurposing. Furthermore, there have been limited holistic investigations regarding how RDNPs can effectively support repurposing. We launched the Repurposing of All Drugs, Mapping All Paths (ROADMAP) Project to characterize the state of rare disease drug repurposing, define a data-driven drug repurposing (DDDR) approach, and identify repurposing optimization opportunities for RDNPs.^27^

## Methods

**Figure 1.**
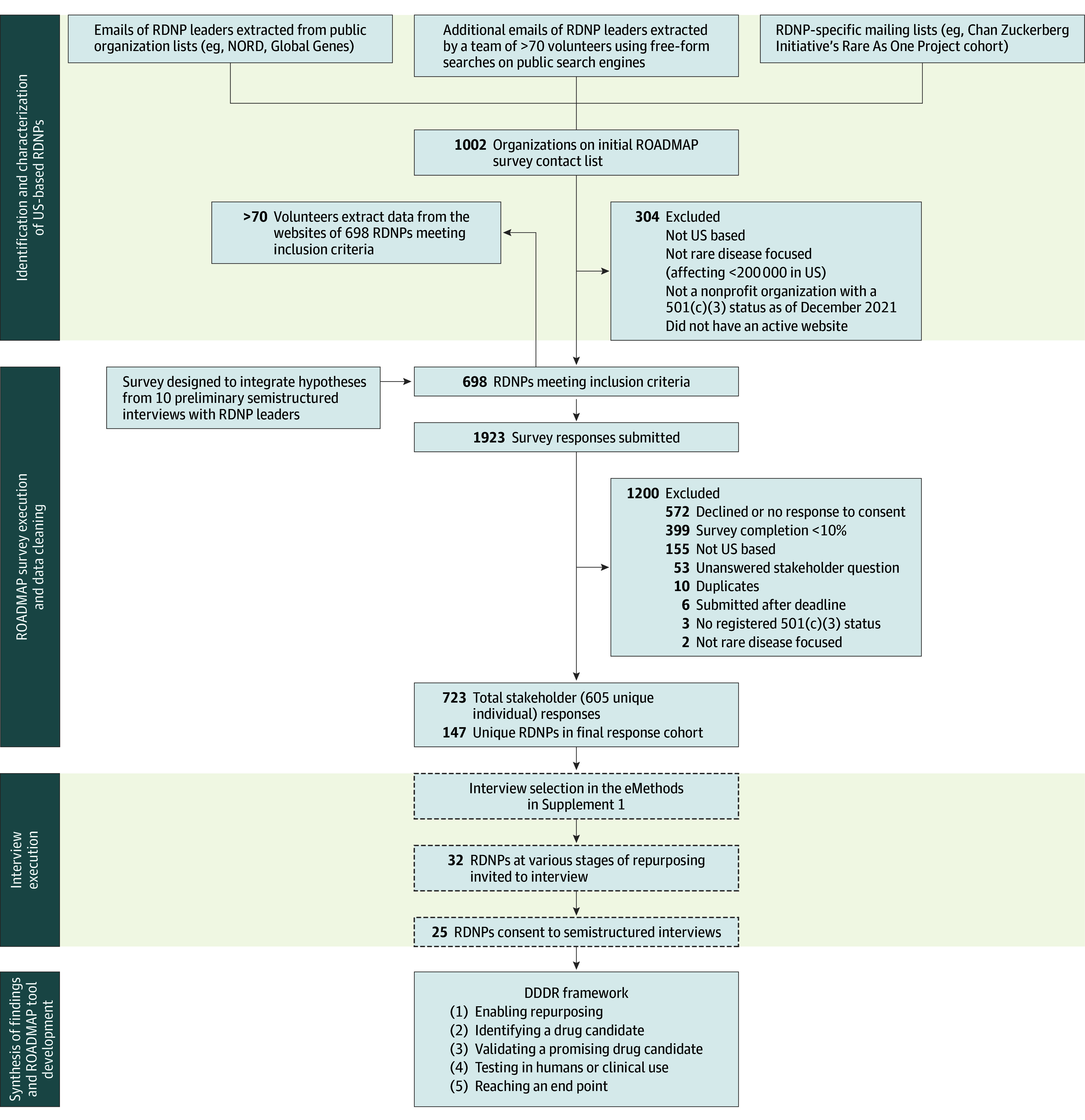
Identification of US-Based Rare Disease Nonprofit Organizations (RDNPs) and the Repurposing of All Drugs, Mapping All Paths (ROADMAP) Project Study Execution The ROADMAP survey was administered to RDNP mailing lists and email addresses from a list of 698 US-based RDNPs that qualified for the study. This list was assembled by a team of more than 70 trained volunteers following extraction from existing lists of RDNPs and searches of public databases. The same team extracted public data from the websites of these RDNPs to characterize the surveyed cohort; 1 author (S.N.) later extracted GuideStar Pro (Candid) data^28^ on all entries. Among ROADMAP respondents, 147 unique, US-based RDNPs were represented, 25 of which participated in hour-long, semistructured interviews. Insights from interviews and survey data iteratively informed the development of a framework for data-driven drug repurposing (DDDR). NORD indicates National Organization for Rare Disorders.

This qualitative study used a mixed-methods analysis of RDNP leaders and their stakeholders that included an electronic, US-based survey deployed from September 29, 2021, to January 6, 2022, and semistructured interviews of participating RDNP leaders. Survey and interview data were synthesized using an exploratory sequential design (Figure 1). The study was approved by the Advarra Institutional Review Board. Written informed consent and permission for interview follow-up were solicited and ascertained at the beginning of the survey. The Standards for Reporting Qualitative Research (SRQR) reporting guideline was followed.

### ROADMAP Survey Design and Execution

The Qualtrics-based ROADMAP electronic survey (eTable 1 in Supplement 1) included 5 sections tailored to 5 stakeholder identities, including RDNP leaders, patients, patients’ loved ones, physicians, and researchers. Respondents were directed to questions affiliated with each stakeholder identity. Question types spanned list, factor or percentage, Likert scale, and free-text questions. To develop survey questions, we conducted hour-long semistructured interviews with 10 RDNP representatives to posit hypotheses relating to features, resources, and support mechanisms that may influence successful repurposing. Hypotheses derived from authors’ past repurposing experiences were also included.

Since no comprehensive list of US-based RDNPs existed at the study’s launch, a team of more than 70 trained volunteers extracted contact information from existing lists, including members of Global Genes,^29^ the National Organization for Rare Disorders,^30^ and the Chan Zuckerberg Initiative’s Rare As One Project^31^ cohort. The same team expanded this list using free-form searches on public search engines to extract email addresses from RDNP websites. We invited contacts to complete the ROADMAP survey using email and encouraged sharing the survey internally with stakeholders. Rare disease initiatives, groups, and nonprofit organizations of US-based RDNPs (Figure 1 and eTable 2 in Supplement 1) met the following inclusion criteria: (1) US based, (2) registered 501(c)(3) nonprofit organization, (3) focused on 1 or more rare diseases (affecting <200 000 individuals in the US), and (4) managed an active website as of December 2021. Data cleaning is summarized in the eMethods in Supplement 2. To characterize RDNPs, the same team extracted mentions of variables of interest from RDNP websites (eTable 2 in Supplement 1), and 1 author (S.N.) extracted publicly available tax information from GuideStar Pro (Candid).^28^

### Survey Outcomes

The primary survey outcome, repurposing project stage, was categorized ordinally during the analysis as (1) unsuccessful or abandoned; (2) early stage without success or abandonment (early stage); (3) clinical stage without success or abandonment (clinical stage); (4) late stage without success or abandonment (late stage); and (5) clinical success. A successful outcome for study purposes included either FDA approval or off-label use with both evidence of efficacy and patient benefit, including significant symptom reduction, quality-of-life improvement, increase in life expectancy, or relapse prevention. Authors verified FDA approval using the administration’s website.^32^ For off-label drugs, patient benefit was RDNP reported, and evidence of efficacy was verified by authors using PubMed if efficacy was published in at least 1 disease-related end point of a cohort study or a case study or series due to disease rarity. Other stage-specific criteria are included in the eMethods in Supplement 2.

### Interview Execution and Thematic Analysis

Of 58 RDNPs pursuing repurposing, leaders from 32 were invited for 1-hour semistructured interviews. Of these, 25 RDNPs consented, including 34 organizational leaders, and discussed experiences with 75 repurposing projects collectively. Interview selection is detailed in the eMethods in Supplement 2. Interviews were conducted virtually using video conferencing by 1 author (A.K.) and were video and audio recorded. The study interviewer was trained in qualitative research as a graduate student and notably had not worked with RDNPs prior to this study. Interview audio was transcribed with permission using a transcription service (Otter.ai; Otter)^33^ and was manually reviewed by a volunteer to assess accuracy (supported by S.E.B.). The interview guide included open-ended questions regarding project timelines and follow-up questions to elicit further detail based on survey responses.

The thematic analysis was conducted using a pragmatic paradigm in which a complex process, such as repurposing promising drugs, was actively conceptualized chronologically from a solutions-oriented approach. Identified statements were intended to generate themes regarding RDNP involvement and support during the entire chronologic repurposing process. An initial codebook was developed using a grounded-theory approach^34^ of the first 10 semistructured interviews. The codebook was then revised iteratively throughout the coding process. All interview transcripts were coded (by A.K.) using Dedoose, version 9.0.107 software (SocioCultural Research Consultants).^35^ The thematic analysis of all 25 interview transcripts was reviewed by a second researcher (S.N.) to determine if thematic saturation had been reached.

### Statistical Analysis

 Data were analyzed from January 22, 2024, to April 23, 2024. We explored the association between survey variables (eg, both organization-specific and drug- or disease-specific variables) (eTable 1 in Supplement 1) and repurposing project stage. We used 2 random forest models of drug- or disease-specific as well as organization-specific variables to evaluate factor importance toward inferring project stage. Orthogonal significance testing was conducted using Spearman rank correlation, as it is ideal for the ordinal nature of the primary outcome. *P* values in each model were corrected for multiple hypothesis testing using a Benjamini-Hochberg procedure; a 2-sided *P* < .05 indicated statistical significance. Data preprocessing and other significance testing are summarized in the eMethods in Supplement 2.

## Results

### Study Population

While 1002 rare disease initiatives, groups, and nonprofit organizations were initially identified, 698 potential US-based RDNPs (eTable 2 in Supplement 1) met inclusion criteria, of which 147 participated in the ROADMAP study. Among these, 605 unique individuals submitted responses for 723 stakeholder identities, which included 181 RDNP leaders, 340 patients, 170 loved ones, 23 physicians, and 43 researchers. Similar to the 551 nonparticipating RDNPs (non–ROADMAP RDNPs), ROADMAP RDNPs (Table 1) were most commonly focused on 1 rare disease (ROADMAP RDNPs: 101 [68.7%] vs non–ROADMAP RDNPs: 334 [62.4%]; *P* = .12) and were of similar median organizational age (ROADMAP RDNPs: 10 years [IQR, 5-20 years] vs non–ROADMAP RDNPs: 10 years [IQR, 6-21 years]; *P* = .38), geographic distribution (ROADMAP RDNPs: 55 [37.4%], Northeast; 22 [15.0%], Midwest; 36 [24.5%], South; and 34 [23.1%], West vs non–ROADMAP RDNPs: 178 [32.3%], Northeast; 107 [19.4%], Midwest; 147 [26.7%], South; and 119 [21.6%], West; *P* = .37), and median compensated employee count (ROADMAP RDNPs: 0 [IQR, 0-3] vs non–ROADMAP RDNPs: 1 [IQR, 0-3]; *P* = .92) (Table 1 and eFigure 1 in Supplement 2). There were a few notable intergroup differences. ROADMAP RDNPs had a greater annual median total revenue compared with non–ROADMAP RDNPs ($355 390 [IQR, $90 028-$946 108] vs $172 394 [IQR, $7227-$714 624]; *P* < .001). Additionally, compared with 53 of 147 ROADMAP RDNPs (36.1%), only 85 of 551 non–ROADMAP RDNPs (15.4%) were found to mention repurposing initiatives or treatment guidelines, including repurposed drugs, on their website (*P* < .001).

**Table 1.** Organizational Characteristics of US-Based ROADMAP Study Respondents and Nonresponding RDNPs^a^

Organizational characteristic	Study group		*P* value
	ROADMAP RDNPs (n = 147)	Non–ROADMAP RDNPs (n = 551)	
Age, median (IQR), y^b^	10 (5-20)	10 (6-21)	.38
Total revenue, median (IQR), $^c^	355 390 (90 028-946 108)	172 394 (7227-714 624)	<.001
Annual compensated employees, median (IQR)^d^	0 (0-3)	1 (0-3)	.92
Geographic location^e^			
Northeast	55 (37.4)	178 (32.3)	.37
Midwest	22 (15.0)	107 (19.4)	
South	36 (24.5)	147 (26.7)	
West	34 (23.1)	119 (21.6)	
No. of rare diseases of focus			
1	101 (68.7)	344 (62.4)	.12
Set	46 (31.3)	207 (37.6)	
Founder type^f^			
Patient	38 (25.9)	160 (29.0)	.39
Loved one	90 (61.2)	323 (58.6)	.52
Physician and researcher	22 (15.0)	92 (16.7)	.57
Mention of drug repurposing on RDNP website^g^	53 (36.1)	85 (15.4)	<.001

Abbreviations: RDNP, rare disease nonprofit organization; ROADMAP, Repurposing of All Drugs, Mapping All Paths.

^a^ Data are presented as No. (%) of RDNPs unless indicated otherwise. The Kolmogorov-Smirnov significance test was used for differences in median age, total revenue, and annual number of compensated employees. χ^2^ Goodness-of-fit significance tests were used for categorical variables, including geographic location, number of rare diseases of focus, individual founder types (not mutually exclusive), and mention of repurposed drugs on RDNP websites.

^b^ Based on age in 2023 relative to founding year reported in GuideStar Pro (Candid).^28^

^c^ Based on the latest GuideStar Pro^28^ Internal Revenue Service report as of the 2022 fiscal year.

^d^ As reported in GuideStar Pro,^28^ last filed in 2022.

^e^ RDNP location from GuideStar Pro^28^ tax reports, categorized by the 4 US geographic regions as defined by the Centers for Disease Control and Prevention.

^f^ Categories are not mutually exclusive. RDNPs were included if at least 1 cofounder was in the subcategory. Identities were extracted by a team of trained volunteers. Some RDNPs had founders of other and unknown identities within this broader cohort.

^g^ Based on mention of repurposed drugs in treatment guidelines or initiatives qualified for manual extraction by a team of trained volunteers. Absence of observed mention on RDNP websites by a trained volunteer team does not equate with absence overall.

After characterizing participating RDNPs, we sought to understand the state of repurposing efforts. Among 147 ROADMAP RDNPs, 54 (36.7%) reported that drug repurposing was an activity of focus. Of the 138 RDNPs specifically identifying their involvement in repurposing, 58 (42.0%) described supporting repurposing for the investigated rare disease. Of the ROADMAP RDNPs, 40 (27.2%) reported 94 drugs of focus (76 unique) in various stages of repurposing, 91 of 94 (96.8%) reportedly had RDNP support during this process, 13 (13.8%) had been abandoned, and 23 drugs (24.5%) achieved a successful clinical milestone, including 5 that received FDA approval and 18 that were used off label with evidence of clinical benefit. Of RDNPs pursuing repurposing, 19 of 58 (32.8%) reported repurposing multiple drugs, ranging from 2 to 7 projects overall.

### Repurposing Project Outcomes

Next, authors leading quantitative analyses (S.N. and J.Z.) evaluated the association of organization-specific and drug- and disease-specific variables (eTable 1 in Supplement 1) with successful repurposing outcomes. Models ranked each factor by relative Gini importance (eFigure 2A and B in Supplement 2). Between both models, 7 variables had both high Gini importance and ordinal statistical significance (eTable 3 in Supplement 2): (1) RDNPs supporting patient recruitment into clinical trials (Gini importance, 3.90; ρ = 0.50; adjusted *P* < .001), (2) RDNPs providing nonfinancial research support (eg, patient samples or data) (Gini importance, 0.69; ρ = 0.33; adjusted *P* = .02), (3) RDNPs reporting a greater number of research methods to identify drug candidates (Gini importance, 1.51; ρ = 0.40; adjusted *P* = .003), (4) RDNPs identifying more promising drugs for their rare disease of focus (Gini importance, 1.76; ρ = 0.42; adjusted *P* = .003), (5) RDNPs reporting patient education in their top 3 activities of focus (Gini importance, 1.05; ρ = 0.36; adjusted *P* = .01), (6) RDNPs fundraising using employer matching (Gini importance, 1.78; ρ = 0.47; adjusted *P* < .001), and (7) RDNPs fundraising from a family foundation (Gini importance, 0.78; ρ = 0.36; adjusted *P* = .01).

Five of 7 variables were supported by orthogonal evidence from qualitative interviews. However, the type of fundraising (eg, employer matching) was not explicitly delineated in interview themes or RDNP accounts. The same 5 variables were of nominal significance when considering success outcomes as binary (successful or unsuccessful) (eTable 3 in Supplement 2).

### DDDR Framework

**Figure 2.**
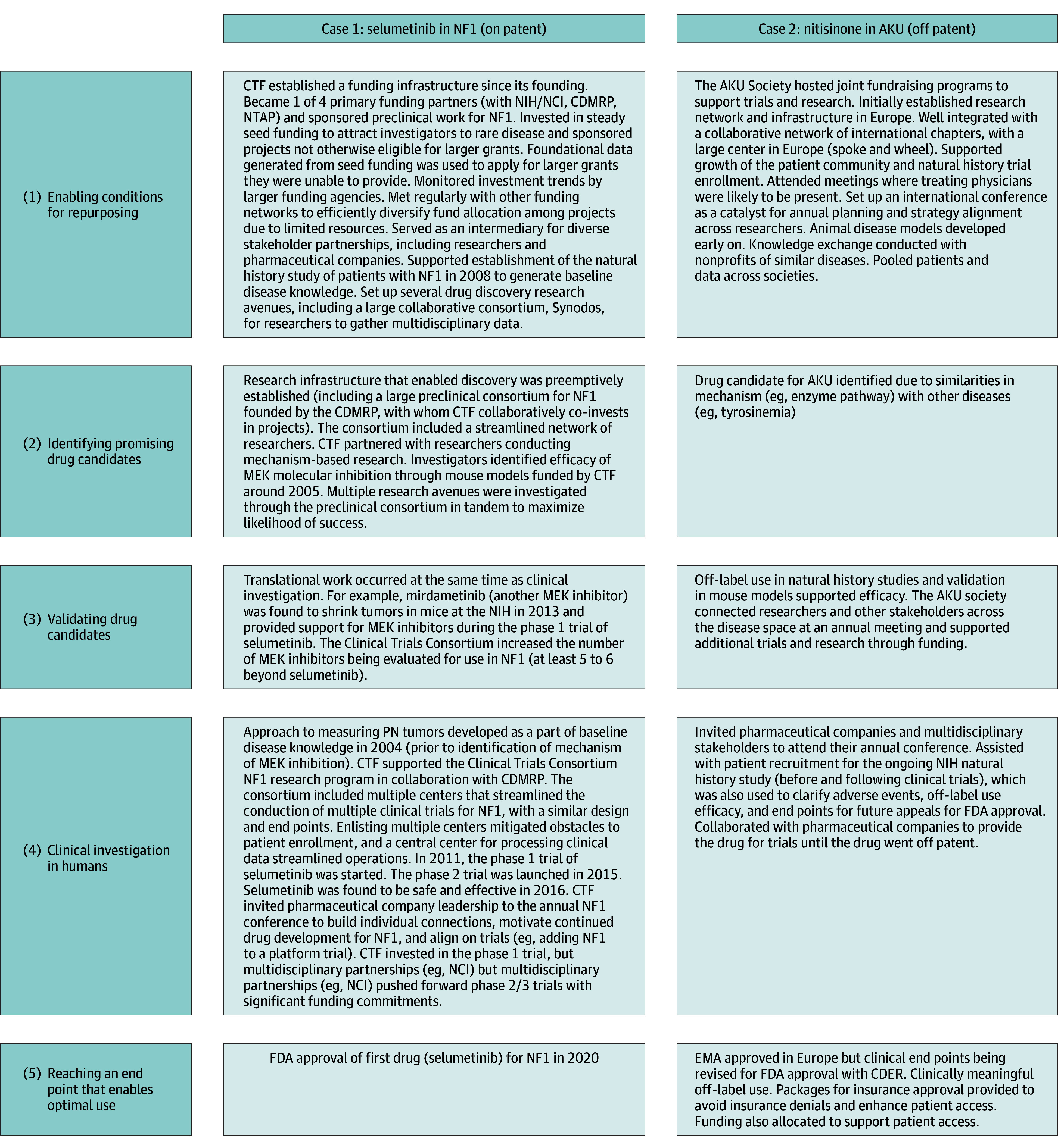
Case-Based Applications of the Data-Driven Drug Repurposing (DDDR) Framework Abbreviated timelines of cases of rare disease repurposing, including the use of selumetinib in neurofibromatosis type 1 (NF1) and nitisinone in alkaptonuria (AKU), are shown. Each case highlights unique paths and considerations while repurposing a drug and the importance of drug and disease profile factors (eg, generic vs on-patent drugs, previous regulatory standards for rare disease). These paths are mapped to the themes and stages comprising a DDDR framework, conceptualizing rare disease nonprofit organization–supported repurposing experiences. CDER indicates Center for Drug Evaluation and Research; CDMRP, Congressionally Directed Medical Research Programs; CTF, Children’s Tumor Foundation; EMA, European Medicines Agency; FDA, US Food and Drug Administration; MEK, mitogen-activated protein kinase kinase; NCI, National Cancer Institute; NIH, National Institutes of Health; NTAP, Neurofibromatosis Therapeutic Acceleration Program; PN, plexiform neurofibroma.

Interviewee characteristics are included in Table 2. Among 34 interviewees representing 25 RDNPs, 23 were female (67.6%), and 11 were male (32.4%). The median age of the RDNPs was 15 years (IQR, 6-19 years), and the median revenue was $670 719 (IQR, $193 587-$1 830 890). Five chronologic repurposing stages (Table 3 and eTable 4 in Supplement 2) thematically emerged during interviews with RDNP leaders. These included (1) enabling drug repurposing, (2) identifying a drug therapy, (3) validating a drug therapy, (4) clinical use and testing, and (5) reaching an optimal end point for clinical practice. Across broader stages, specific subthemes emerged. Stages and themes; subthemes; illustrative quotations; and proposed actions for RDNPs, external bodies, and policymakers are included in Table 3. Despite thematic similarities across RDNPs, repurposing paths were diverse and nonlinear. Not all spanned the same length of time, and, notably, some stages were skipped for a specific project or were not otherwise directly supported by RDNPs. Themes 6 (roadblocks and challenges) and 7 (opportunities or recommendations from RDNPs) are also identified in Table 3. Case summaries of 2 drug–disease pairs (Figure 2) are referenced to exemplify unique paths depending on drug and disease profiles.

**Table 2.** Characteristics of RDNP Leader Interview Participants^a^

Characteristic	ROADMAP RDNPs
**Individuals (n = 34)** ^b^	
Sex	
Female	23 (67.7)
Male	11 (32.4)
**RDNPs (n = 25)**	
Age, median (IQR)^c^	15 (6-19)
Total revenue, median (IQR), $^d^	670 719 (193 587-1 830 890)
Annual compensated employees, median (IQR)^e^	1 (0-7)
No. of rare diseases of focus	
1	20 (80.0)
Set	5 (20.0)
Survey-reported drug repurposing projects^f^	
Successful	12 (48.0)
Abandoned or unsuccessful	8 (32.0)
Not yet meeting aforementioned outcomes	15 (60.0)

Abbreviations: RDNP, rare disease nonprofit organization; ROADMAP, Repurposing of All Drugs, Mapping All Paths.

^a^ Data are presented as No. (%) of individuals or RDNPs unless indicated otherwise.

^b^ The distribution of roles of participants included: executive director, director of operations, cofounder, president, chief executive officer, scientific director, director of research operations, director of research strategy, vice president, and advocacy research coordinator.

^c^ Based on the age in 2023 relative to founding year reported in GuideStar Pro (Candid).^28^

^d^ Based on the latest GuideStar Pro^28^ Internal Revenue Service report as of the 2022 fiscal year.

^e^ As reported in GuideStar Pro,^28^ last filed in 2022.

^f^ Classifications as successful and abandoned or unsuccessful are detailed in the Methods.

**Table 3.** Themes, Subthemes, Illustrative Quotations, and Opportunities From RDNP Interviews^a^

Themes and subthemes	Illustrative quotations	Opportunities	
		RDNP	External collaborator or policy
**(1) Enabling drug repurposing**			
(a) Funding support	“As a nonprofit organization...we don’t have steady income from anywhere, so we have to go out and raise the funds. So, we have focused most of our efforts on joint fundraising programs to support the program that was taking place in [country name] and was successful...the drug was approved in [continent]. And, so, that…helped to lay the groundwork for us, where they had a population that was easily accessible.” (RDNP 2)	Building a scientific or medical advisory board for strategic planning Establishing relationships with all key stakeholders in the space (eg, researchers, companies) Fundraising for key research projects and strategic allocation to obtain foundational data for larger grants across funding networks to diversify coverage Patient engagement and surveys and determining patient-centric repurposing needs Collating existing data (eg, regulatory precedents for disease) Establishing infrastructure for nonfinancial research support (eg, anticipatory collection and storage of patient samples, establishing natural history studies) Annual conferences to align strategies among stakeholders, find pharmaceutical company champions and a streamlined physician and researcher network	Government support in establishing a well-integrated, streamlined rare disease research consortia Increased funding toward rare disease-specific repurposing projects Clinician-driven and researcher-driven repurposing: active search for and early partnership with RDNPs
(b) Research support	“The biggest thing is to set up centralized research capabilities so that you don’t have to continually find academics to recreate the same capabilities over and over…. We needed to create models and model repositories and a biobank for many different purposes…none of this would have been possible until cell lines existed.” (RDNP 6)		
(c) Partnering and collaboration	“We brought together experts from all over the world…people that were doing cell models, animal models, transcriptomic, proteomic [analyses]…. Before I came to the foundation, [we also had] an initiative…which was a group of labs we [work with to] bring drugs to the clinic that have shown value in a preclinical model. With [a group of] about 5 labs, who would work together [to] select certain drugs, they [would] test them in their preclinical model and bring them to the clinic. So that was the first attempt to look at drugs for [disease] repositioning, repurposing, or [drugs anywhere] in clinical development…. Then those drugs were brought to [clinical trials].” (RDNP 5)		
(d) Patient support	“We’re trying to be that support system for our patient community as well. We’ve been collecting information and guiding families. For example, we worked with the manufacturer and…[identifying person’s] geneticist to get compassionate-use approval for a drug, [repurposed drug name], that had preclinical data. While the trial didn’t lead to significant improvements, it was important for us to be involved in ensuring families had access to potential treatments and understood the options available. We know once we share any new information, families rush to figure out how to access it for their child. So, we need to provide clear guidance on dosing, side effects, and how to obtain the drug.” (RDNP 14)		
**(2) Identifying a drug therapy**			
(a) Targeting mechanisms related to disease	“[In our case], the search for a drug didn’t start with the intent that it has to be a repurposed drug…[it] started in the context of…let’s understand the molecular pathogenesis. And based on that, we can see which targets seem right…. And, as it so transpired, when the discovery of the genetic mutation and the main pathway that mutation was targeting, there was a ready-made drug that already existed.” (RDNP 16)	Systematically tracking off-label use Sponsoring key translational research studies among collaborators Integrating a researcher network and multidisciplinary disease data (eg, existing nonclinical, clinical, and population data) through conferences or patient-centered challenges Early mapping of regulatory pathway elements and potential expedited pathways to identify data gaps Use of translational and in silico computational tools (eg, REMEDi4ALL)	Governmental programs to formalize data standards for on-patent and off-patent repurposed drugs Clinicians and researchers: engage patient organizations from early nonclinical and discovery phases to facilitate multidisciplinary collaboration
(b) Drug-centered screening approaches	“[After the previous project], we initiated a new zebrafish grant…[with] the hope that it could be used for drug screening. So, [using] a library of FDA-approved drugs, [we are] looking for rescue of [identifying pathologic deficit] in those fish [with] a private company.... We are funding a characterization of the model and [the testing of] several drugs on it...any drug that’s been identified in the literature as potentially beneficial to [disease name].” (RDNP 8)		
(c) Data from initial off-label and human use	“[Research institute name] is doing some basic/translational science on that, [but] we kind of worked in reverse order. We took the real-world data from our population and then returned that information when we observed the changes in our children after taking [drug name]. Using the real-world data is when we discovered the [identifying mechanism] that [identifying disease gene] controls in the [identifying organ], which is [pathologically] depressed. [We put] 2 and 2 together [from there] because [repurposed drug name] also has an indication [in] [another disease with a similar mechanism].” (RDNP 25)		
(d) Looking at similar diseases	“An enzyme process that happens in [our disease] [was] very similar to [another disease]…[and] so then [the] idea [was to] study the parallels—it’s the same [identifying mechanism]…. And it worked amazingly well…. It was discovered that when patients took [repurposed drug], it reduced [disease pathological substance] by 95%,...[and] that started the clinical trials, both in North America and across Europe.” (RDNP 2)		
(e) Computational approaches	“We worked with the [academic institution] [animal model] lab on computational models,…and they did a computational screen to identify [repurposed drug candidate] as a potential candidate for [disease].” (RDNP 22)		
**(3) Validating a drug therapy**			
(a) In vitro validation	“Once the central discovery of [pathway class name] pathway was discovered, it was relatively easy to make the connection that there is a preexisting drug…this was very rapidly tested in a preclinical model, which showed the desired, expected results…the gene discovery and the fruit fly experiments were serendipitous…once the linkage of the gene mutations to [pathway name] pathway was discovered, that first bit of preclinical science after that was funded by [our RDNP].” (RDNP 16)	Overlapping opportunities listed under theme 2	Overlapping opportunities listed under theme 2
(b) In vivo validation	“So, there were 2 approaches that were taken…. One of them was with mouse iPSCs [induced pluripotent stem cells], and the second was in worms…. And they were able to see that those aggregates were basically resolved, with the administration of the [repurposed drug]…[but the screen] was targeted…20 or less…[since] they had a mechanism that they were targeting.” (RDNP 23)		
(c) Clinical evidence	“Some of our researchers discovered the [identifying disease-specific mutation]…. After that, there was a doctor…who tried a [drug class targeting that mutation] under managed access and had some success…. And so he started this drug [in a patient with end-stage disease] and [the patient] had a pretty immediate response…. After that happened, [he] published…a case [series]…. That was the second hit that led us to what came next…. People in the US started applying for compassionate use and getting on the medication, [and] there was…enough movement and action that [pharmaceutical company conducted] a retrospective study of all the people who have taken the medicine via compassionate use…between preclinical and clinical [studies].” (RDNP 7)		
**(4) Clinical use and testing**			
(a) Clinical trial investigations	“[The repurposed drug candidate trial was first conducted as] phase 0, phase 1…it was positioned as a safety study because it was the first time in this population.” (RDNP 23)	Assisting with patient recruitment into trials Supporting patient-centric design and conduction of trials Establishing longitudinal natural history registries During collation of disease data, early collaboration with pharmaceutical companies and regulatory bodies to determine appropriate end points, including potential tools (eg, C-PATH)	Governmental establishment, support, and growth of pilot programs to support patient-centric establishment of rare disease end points (eg, RDEA pilot program) Data harmonization of off-label use and clinical data and integration with EHRs Clinicians and researchers: report case studies of off-label use through formal reporting systems (eg, CURE ID)
	“Because it was a drug-repurposing effort, they were able to move right into a large phase 2, 3 study…the hope is that this is going to be approved on the strength of a single clinical trial, which is obviously far accelerated from a traditional drug development pathway.” (RDNP 19)		
(b) Observational studies and off-label use	“[Pharmaceutical company name] applied for approval [of the repurposed drug candidate] through the [identifying regulatory body]. And the [regulatory body] granted them conditional approval, with the specification that the long-term safety and efficacy of this drug [need] to be studied in a prospective manner. And that’s where we, as it so happened around the same time, already had an existing prospective longitudinal natural history registry. And we were able to collaborate with [pharmaceutical company name] and tell the [regulatory body] that we will use the data from this registry and give it to [pharmaceutical company name] to use that data to help with full [regulatory body] approval. And after 4 years of longitudinal data collection, we were actually able to obtain full [regulatory body] approval just a few months ago.” (RDNP 16)		
**(5) Reaching an optimal end point for clinical practice**			
(a) FDA approval	“[Over a 4-year time period], [everyone prepared] for FDA submission, and [we were] going through the submission process. [During this period], the drug went off patent, and the [pharmaceutical company] interest…was understandably not so great. So, citizens petitioned, working through a lot of just incredible, heroic efforts…everyone coming together to get this across the line for FDA approval…. This [citizen] petition was to change the product label for [drug name] in the absence of an application from the manufacturer,…but [ultimately, we were able to do so using the preferred route with the manufacturer after FDA discussions with the company].” (RDNP 16)	Setting outcome and optimal end points for repurposing at the beginning based on patient-centric repurposing goals Anticipating access issues and creating holistic access plans early if nonapproval end points are set	Establishment of governmental and nongovernmental financing models and business models for on-patent and off-patent repurposed drugs
(b) Alternative end points and off-label use	“Over the last decade, people have been using [repurposed drug] off label…. [For] patients, it’s been a lifesaver. Now there’s a population of patients it doesn’t work for at all, or it works, and then it stops,…[but] people are still using it off label. Ironically, it’s paid by Medicare and covered, and [that was done] without a phase 2/3 trial.” (RDNP 11)		
**(6) Roadblocks and challenges**			
(a) Lack of previous involvement in, and/or knowledge about repurposing	“So, we have candidates…being tested at [another organization] in [identifying cell type],…[but] once we have this information,…[our] small team has to try to figure out what we’re going to do with it, how do we get this out there. [And], you know, a lot of what we’re trying to do as an organization is [to] provide that information to them. So, for us, to have the expertise and the guidance [would] help us get things to the next level….” (RDNP 14)	Using recently released guidebooks, tools, and checklists to establish a repurposing roadmap specific to the profiles of the drug and disease	Build a central US platform to support public repurposing projects across the value chain, including methodologic, financial, legal, regulatory, and intellectual property aspects Establishing funding models and networks to support repurposing, particularly off-patent drugs
(b) Lack of knowledge about disease or drug–disease relationship	“One of the problems with [the disease being tested] is that there is really no route to approval right now, other than these very difficult studies with biopsies. And, I mean, we haven’t seen a drug approved. So, we don’t really have good end points in [the disease being tested].” (RDNP 20)		
(c) Lack of physician or researcher collaboration and data sharing	“The [disease name] field used to be extremely siloed…and has [these] data, [where] I need samples, and no one shares…. There are 15 other [disease name] registries worldwide…. So, you think, ‘Okay, you’ll have your 30 patients in your registry,’ but some of those could also be in this other registry, or you’re missing this information that can be useful for your patients…. I spent 6 months trying to get cell lines from [foreign country] that we didn’t end up getting because they have such strict rules about sharing samples…. So, imagine all the patient data….” (RDNP 12)		
(d) Lack of pharmaceutical company support and incentive	“We approached [pharmaceutical company], and they told us ‘no.’ We tried for 2 years, actually…. And then more and more physicians started dipping their toe in…. [It was still a] ‘no go.’… [Another pharmaceutical company] was not interested…[and] didn’t give a lot of detail…[even when] we had our scientists, we had our physicians, we had patients [weighing in].” (RDNP 11)		
(e) Difficulties with regulatory bodies and approval issues	“[Identifying regulatory body] would not pass it without a full phase 3 clinical trial with a placebo arm. And so that’s where we’re at now…. It’s so hard because especially with something like [topical repurposed drug], the side effects are just nothing…it’s…frustrating to meet roadblocks from the regulatory side.” (RDNP 18)		
(f) Access issues and off-label concerns	“Because it is off-label use…it’s just a battle to get approved for coverage for it…. Every insurance company negotiates their own price…we’ve got some people paying $27 a month, some people paying $2500 a month.” (RDNP 24)		
(g) Difficulties with clinical trial design, recruitment, and conduction	“The trial itself has been extremely challenging, frankly…. There was a very small trial [and was] positioned as a safety study with…10 patients. And…it was the first time that [the repurposed drug] had been administered in this population. So, even though [the repurposed drug candidate] had been FDA approved,…it was still the first time…and not having ever done this before [was an] issue,…[and we had limited] clinical end point information.” (RDNP 23)		
(h) Lack of patient involvement or support	“There is a large community of clinicians and researchers in this space, and there have been for decades, which is fantastic…. But because of that, there are so many players in this space, and the role that we have as a patient organization historically hasn’t been as clear…. [In fact], the treatment guidelines are being updated right now,… [but] no patient is involved in the guidances yet…. [Whether the drug will be recommended in US guidance is] a particularly emotional topic because many of the people who report a response [to the drug] are children. And some of the responses appear to be quite dramatic; they go from being very sick, you know about to have a [identifying corrective procedure], to perfectly normal....” (RDNP 20)		
(i) Rare disease-specific obstacles and drug-specific patent challenges	“[Name of generic drug] is very well tolerated. It’s easily accessible, and it’s cheap…. So there’s very low motivation to do any more work on this one…. No one’s thinking, ‘Oh, well, I need to get it indicated so that I can get it reimbursed.’ So there’s very low motivation to do any more work on this one….” (RDNP 4)		
**(7) Opportunities or recommendations from RDNPs**			
(a) Patient recruitment and patient engagement at all steps of repurposing, including setting end points	“More than 60% of patients in [our] surveys are taking it,...and it’s higher in Europe because it’s much more widely accepted.... Probably the most frustrating thing [about lack of formal recommendation/approval] for patients is they say, ‘When I go off [off-label drug], I feel worse. I feel better when I go back on.’ [I think] finding evidence-based ways to document quality-of-life issues [is important] because that’s why [off-label repurposed drug] seems to be used so much…. It seems to really help quality of life, even though it hasn’t been proven to conclusively change disease course.... The treatment guidelines are being updated right now. And they differ, depending on the society issuing them.” (RDNP 20)	Establishing forms of systematic data collection, including clinical evidence and off-label use Crowdsourcing research and research ideas, including lists for hypotheses and unique cases Leveraging conferences as an avenue to find internal pharmaceutical company champions for rare disease Collaborative data-sharing, data harmonization, and an active combination of multidisciplinary methods across researchers (eg, through conferences, funded challenges) Facilitating access to efficacious therapies using prepared insurance packages of drug efficacy Exploring alternative models for clinical trial execution (eg, platform, n = 1)	Governmental establishment of patient-driven committees to provide feedback on disease end points and growth of existing opportunities Increased consideration and adoption of quality-of-life measures and other patient-driven measures in end points
	“We have patient families who’ve seen really significant reduction in symptoms, so [repurposed drug] is definitely helping,…[and we have a lot of] data showing safety and efficacy…. It is being used widely off label [now]…. [But, the] FDA originally rejected the [repurposed drug] application on safety concerns…. [In addition to collecting longitudinal safety data through our registry], we’re trying to set up a patient-listening session with the FDA for the fall so that they better understand what living with [disease name] is like and the patient and family care burden…. If this disease goes on unmitigated, then the consequences are dire. So, we are willing to take on these risks [from the repurposed drug and are] trying to very clearly understand so that we can give our children opportunities that they wouldn’t otherwise have.” (RDNP 1)		
(b) Leveraging collaboration with pharmaceutical companies and other multidisciplinary research partners	“[RDNP website name] was helpful in bringing researchers together…. There was really no funding; it was more of a networking thing, connecting researchers together once there was a hit…. As part of one of my broader research collaborations,…there’s a [government]-funded rare disease clinical research network associated with [our RDNP]. And that rare disease clinical research is funding clinical trials now.” (RDNP 4)		
(c) Establishing forms of systematic data collection and crowdsourcing research and research ideas	“We started a large collaborative consortium…. I call it the football model.… First, patients contact us [with a research question]…. [And] the whole idea is we bring together experts from all over the world [to address the question]. So, we really needed to have people with very different skill sets that are part of the…team. And, the ultimate client of what we’re doing has to be part of [the] consortium…so we have patients in the consortium. We say, ‘Okay, you guys, we put [$ amount] on the table,’ [and all researchers] submit something to solve this conundrum.... So, [everyone] came together: people that were doing cell models, animal models, transcriptomic, [and] proteomic studies…. [Repurposed drug candidate] was incredibly powerful [in cell and animal models],…[but the transcriptomic and proteomic studies were also needed] to actually figure out that this molecule was hitting mechanisms that were super relevant for [the disease being tested].” (RDNP 5)		
(d) Exploring alternative models for clinical trial execution	“I think that the future will probably see the [RDNP name] running a platform trial…. It kind of gives us the power, where, otherwise, we’re getting scraps of information.” (RDNP 1)		
	“After [pharmaceutical company manufacturer] made the decision against [submitting materials to the FDA for a label change], you can [conduct a trial] under what’s called an IND exemption…. You don’t have to take the 1700 additional steps that are really expensive and time consuming, that just make your trial way, way more expensive.” (RDNP 3)		
(e) Facilitating access when alternative end points set	“By the end, we had a really good package put together that addressed all of the information. And, so, [after] sharing that with other physicians, there’s been no one that’s been denied this package to use it off label…. We push [for coverage using] the [mechanism] because…if you can stop that, then none of those [cost-intensive] things will happen [downstream]. And so that [cost-efficacy] case is made.” (RDNP 2)		

Abbreviations: C-PATH, Critical Path Institute; EHRs, electronic health records; FDA, US Food and Drug Administration; IND, investigational new drug; RDEA, Rare Disease Endpoint Advancement; RDNP, rare disease nonprofit organization; REMEDi4ALL, Repurposing of Medicines 4 All.

^a^ A data-driven drug repurposing framework, including 5 chronologic themes and 2 nonchronologic themes, were extracted and discussed from RDNP interviews. Timelines of repurposing projects varied, and RDNPs did not necessarily support all stages for each project. Repurposed drug candidates also did not always proceed through all stages. A full spectrum of quotations is included in eTable 4 in Supplement 1. From selected themes, multiple opportunities for RDNPs, external collaborators, and governing bodies are available to facilitate rare disease repurposing.

### Theme and Stage 1: Enabling Drug Repurposing

RDNP representatives took diverse steps to secure resources and infrastructure for repurposing projects. This often included subthemes of acquiring key funding, research support, partnerships, and patient support. In practice, while funding studies for selumetinib (Figure 2), for example, the Children’s Tumor Foundation optimized repurposing with limited resources by using the following specific strategies: regularly meeting with other existing funding agencies or nonprofit organizations for neurofibromatosis type 1 to strategically allocate limited funds across projects, monitoring federal agency research investment trends, and providing steady seed funding that provided foundational data for other larger grants. Other strategies and infrastructure beyond this case (Table 3) included scientific and medical advisory boards, natural history studies, registries, research agendas, systematically tracking off-label use, and collection and provision of patient data and samples. Partnerships for successful projects were extensive, including researchers (eg, data generation, trial planning), RDNPs (eg, joint funding applications, pooling patients and resources), and pharmaceutical companies (eg, drug donations, expanding platform trials, and navigating access issues).

### Themes and Stages 2 and 3: Identifying and Validating Drug Therapies

RDNPs supported various methods to identify promising drug candidates, including (1) conducting preclinical and translational research related to mechanism and pathogenesis; (2) drug-centered screens; (3) analyzing data from similar diseases, mechanistically or phenotypically; (4) identifying patterns from off-label use; and (5) using computational approaches. In case 1 (Figure 2), the Children’s Tumor Foundation invested in the first preclinical mouse model supporting the efficacy of mitogen-activated protein kinase kinase (MEK) inhibition in neurofibromatosis type 1; this research was enabled by integrated networks and infrastructure constructed by external bodies and collaborators (eg, the Preclinical and Clinical Trials Consortium founded by the Congressionally Directed Medical Research Programs). RDNPs supported drug candidate validation (Table 3) through in vitro and in vivo preclinical evidence and prospective and retrospective clinical evidence. Evidence required for clinical testing and regulatory approval varied, influenced by disease rarity, extent of drug use (eg, already used for years off label, previous natural history studies), and drug–disease risk profile.

### Theme and Stage 4: Clinical Use and Testing

RDNPs facilitated clinical translation of promising drugs through trials, off-label use, and/or observational studies. Evidence requirements varied considerably. Traditional pathways, beginning with phase 1 trials, were sometimes required (case 1). This often occurred when the drug differed sufficiently in dose or formulation or varied compared with other members of its drug class (eg, MEK inhibitors for other cancers) or if the new disease population differed significantly compared with its initial indication. On the other hand (case 2: the Alkaptonuria Society repurposing off-patent nitisinone in alkaptonuria), RDNPs could take advantage of expedited pathways and transition to a phase 2 or 3 trial, complemented by supplemental clinical data. These differences were often associated with drug-risk profile, patent status, and targeted milestone (eg, FDA approval). Successful RDNPs (Table 3) also assisted with trial design, conducted natural history and observational studies, and established uniform clinical end points early.

### Theme and Stage 5: Reaching an Optimal End Point for Clinical Practice

Determining appropriate clinical milestones for patient use was a multifactorial decision dependent on patient needs; the rare disease; and drug safety, risk, and efficacy profiles. Some RDNPs (case 1) chose to pursue FDA-approved indication expansion; however, some instead elected to publish studies and advocate for off-label use and/or adoption in treatment guidelines. This choice often stemmed from the significant time and expense associated with FDA submission, which were generally implausible without backing from the manufacturer authorized to request label modifications. Particularly with off-patent drugs (case 2) in rare diseases, there is often insufficient incentives for pharmaceutical companies to submit for FDA approval. Off-label use as a meaningful milestone sometimes included access roadblocks (eg, copay assistance), with RDNPs often compiling market access packages to facilitate insurance appeals.

## Discussion

This qualitative study with a mixed-methods analysis included a cross-sectional assessment of RDNP repurposing and a qualitative framework of repurposing stages, gaps, and opportunities derived from RDNP experiences. While just under half of ROADMAP RDNPs pursued repurposing (42.0%), a substantial number of repurposed drug-disease pairs were supported by this cohort, representing a sizable area of focus for RDNPs.

There were several key findings. For RDNPs, this study provides a qualitative repurposing framework, developed and curated primarily from the experiences of RDNP peers, which included a 5-stage DDDR approach. A comprehensive synthesis of thematic findings is available in an interactive final product, the online ROADMAP tool.^27^ This framework is additive to recent efforts to provide infrastructure for repurposing, including target and product discovery tools (eg, compound and network databases),^36,37,38,39^ clinical evidence platforms (eg, the CURE Drug Repurposing Collaboratory),^40^ regulatory kits (eg, the National Center for Advancing Translational Sciences’ toolkit),^41^ guidebooks and toolkits (eg, the International Rare Diseases Research Consortium’s Orphan Drug Development Guidebook),^42^ and checklists and templates (eg, the START [Stakeholder Mapping, Available Information on the Disease, Resources, and Target] checklist and Patient Value Profile templates).^43,44,45^ Within the context of these tools, this framework uniquely adds insight into peer experiences, including roadblocks and recommendations, and repurposing experiences at all stages, including abandoned projects (eTable 4 in Supplement 2 and Figure 2). As such, this framework can be used alongside the aforementioned tools to enhance planning and execution of a repurposing program, including referencing peer recommendations, hurdles, and missteps. Collectively, these tools and this study’s findings provide a foundation for DDDR, which reframes repurposing as a deliberate, evidence-based endeavor that can be optimized and supported by stakeholders and governmental authorities.

We highlighted several recommendations based on interviews, many of which have low fiscal barriers, including leveraging close collaboration with pharmaceutical companies and stakeholders; establishing forms of systematic data collection and crowdsourcing research and ideas; patient engagement at all steps of repurposing, including trial end points (eg, patient preference studies); and exploring alternative clinical trial models, as well as considering and facilitating market access at an early time point. Some of these factors, like data collection from off-label use, are supported in case studies and literature reviews.^46,47,48,49^ Factors were also identified in the ROADMAP survey. Patient recruitment into promising trials and expending nonfinancial research support are prominent because they are actionable and less financially demanding opportunities for RDNPs. Causation of success cannot be inferred due to the observational nature of the study, and additional work is required to explore these findings.

For policymakers and external collaborators, RDNP narratives provided insight on rare disease repurposing barriers. We highlighted several proposed changes (Table 3) to facilitate systematic repurposing, including establishing a US-equivalent central platform to support repurposing projects at all stages; establishing funding models for repurposed drugs, particularly off-patent drugs; establishing clear repurposing data standards; and increasing patient feedback opportunities for inclusive end points, such as quality of life. For physician-driven and researcher-driven repurposing, we highlighted future synergies and structural changes, including harmonizing clinical data from off-label use from formal reporting mechanisms and engaging patient organizations at an early time point in nonclinical and discovery phases.

From the perspective of health policy, this qualitative study analyzed differential variables associated with successful or unsuccessful repurposing projects, with definitions tied to fulfillment of patient needs. These factors should be validated for future repurposing optimization. Since repurposing outcomes can have a meaningful benefit for patients through on-label or off-label use, this study also highlights the need to address barriers to access, including regulatory and reimbursement challenges. Under the view that repurposing is a data-driven endeavor to be optimized across drugs, a measure is required to assess whether existing drugs meet their theoretical utility across diseases.

### Limitations

This study has several limitations. First, it is possible that not all US-based RDNPs were identified, excluding important perspectives. Second, variable organizational responsiveness and nonprofit lifespans may have contributed to survivorship and nonresponse bias among participating RDNPs. Importantly, assessments of ROADMAP RDNPs vs nonresponders suggest good external validity among numerous confounding variables. However, ROADMAP RDNPs generated higher revenue and were more likely to mention repurposing on their website, which are proxies suggesting greater resources and repurposing experience or knowledge. Despite this, ROADMAP RDNPs discussed financial limitations, which may be understated; however, many proposed repurposing opportunities included low barriers to implementation. Third, while user feedback was collected before dissemination, survey length and complexity may have led to inaccurate or incomplete respondent data. Fourth, while this provided a snapshot in time of nonprofit-supported repurposing, systematic longitudinal data collection will be necessary to validate findings and accurately assess repurposing progress.

## Conclusions

The findings of this qualitative study, which included a mixed-methods analysis of RDNP repurposing, suggest that several factors of RDNP-supported repurposing were associated with successful outcomes. The study provided a DDDR framework to facilitate RDNP repurposing and proposes several actionable changes to optimize repurposing for external collaborators, policymakers, and RDNPs.

## Notes

Supplement 1. **eTable 1.** ROADMAP Survey Questions, Data Cleaning, Preprocessing, and Assigned Variables **eTable 2.** List of US-Based RDNPs Meeting ROADMAP Inclusion Criteria and Organizational Demographics

Supplement 2.
eMethods
**eFigure 1.** Distribution of Organizational Demographics of ROADMAP Survey Respondents Compared With the Broader US-Based RDNP Landscape **eTable 3.** Drug-Disease Pair-Specific and Organization-Specific Variables Associated With Successful Repurposing Project Outcomes **eFigure 2.** Random Forest Models of Drug-Disease Pair-Specific and Organization-Specific Variables Associated With Successful Repurposing Project Outcomes **eTable 4.** Themes, Subthemes, and Illuminating Quotations From ROADMAP Rare Disease Nonprofits

Supplement 3.
Data Sharing Statement
