# Supplementary material for: Rare Disease Drug Repurposing
Source: JAMA Netw Open. 2025 May 5;8(5):e258330. doi: 10.1001/jamanetworkopen.2025.8330 (PMC12053540; doi:10.1001/jamanetworkopen.2025.8330)
Supplement: Supplement 2. — eMethods eFigure 1. Distribution of Organizational Demographics of ROADMAP Survey Respondents Compared With the Broader US-Based RDNP Landscape eTable 3. Drug-Disease Pair-Specific and Organization-Specific Variables Associated With Successful Repurposing Project Outcomes eFigure 2. Random Forest Models of Drug-Disease Pair-Specific and Organization-Specific Variables Associated With Successful Repurposing Project Outcomes eTable 4. Themes, Subthemes, and Illuminating Quotations From ROADMAP Rare Disease Nonprofits [file jamanetwopen-e258330-s002.pdf]

Supplemental Online Content

Nijim S, Korsunska A, Zinski J, et al. Rare disease drug repurposing. *JAMA Netw Open*. 2025;8(3):e258330. doi:10.1001/jamanetworkopen.2025.8330

eMethods
eFigure 1. Distribution of Organizational Demographics of ROADMAP Survey Respondents Compared With the Broader US-Based RDNP Landscape
eTable 3. Drug-Disease Pair-Specific and Organization-Specific Variables Associated With Successful Repurposing Project Outcomes
eFigure 2. Random Forest Models of Drug-Disease Pair-Specific and Organization-Specific Variables Associated With Successful Repurposing Project Outcomes
eTable 4. Themes, Subthemes, and Illuminating Quotations From ROADMAP Rare Disease Nonprofits

This supplemental material has been provided by the authors to give readers additional information about their work.

eMethods

Survey Outcome Definitions and Classification Decisions
For analytical purposes: (1) "early-stage" included steps preceding formal clinical investigation (e.g., laboratory testing, securing funding/data, or early off-label use without knowledge of efficacy); (2) "clinical-stage" included active investigation in human trials/studies; and (3) "late-stage" included post-trial steps, such as data analysis and FDA submission. "Unsuccessful" repurposing outcomes included projects reportedly abandoned or otherwise not further pursued (e.g., efficacy/safety concerns).

Of note, stage did not preclude qualifying for "success"/"abandonment" due to diversity of reported repurposing paths (e.g., "abandonment" during late stage, disseminated off-label use with "successful" community benefit during clinical stage). Projects meeting more than one stage's criteria (e.g., finalizing clinical trials and preparing FDA documents) were allocated to the later stage if they did not already meet a "success"/"abandonment" endpoint. A "successful" outcome for study purposes included either (1) FDA-approval or (2) off-label use with both "evidence of efficacy" and "patient benefit", including significant symptom reduction, quality of life improvement, increase in life expectancy, or relapse prevention. Authors verified FDA-approval using Drugs@FDA[31]. For off-label drugs, "patient benefit" was RDNP-reported and "evidence of efficacy" was verified by authors using PubMed if efficacy was published in at least one disease-related endpoint of a published cohort study or case series/study, due to disease rarity.

Survey Data Cleaning
Of the initial 1,923 entries, 1,200 were removed due to being answered by RDNPs that did not meet inclusion criteria, incompleteness, or as a result of deduplication, resulting in a final dataset of 723 total (605 unique individual entries representing RDNP leaders and other stakeholders from a spectrum of 147 RDNPs). We performed data cleaning of ROADMAP survey data, sequentially removing entries if: the RDNP was not a registered 501(c)3 (n=3); not rare disease-focused (n=2); not US-based (n=155); submitted after deadline (n = 6); survey completion <10% (n=399); and, subsequently, if the participant declined consent or did not answer the consent question (n=572), did not answer any questions in the stakeholder section (n=53), or was a duplicate (n=10). We applied exclusion criteria sequentially in the aforementioned order; thus, absolute counts for each subsequent exclusion criterion are underestimated. If multiple RDNP leaders from the same organization submitted an entry, we selected the RDNP leader submission with the most comprehensive data. We rectified data irregularities, including instances such as multiple entries from a single participant, and we updated the data when new information was received, such as FDA approval of a drug. All manually-entered text from survey free responses were re-classified if applicable and standardized across submissions. All data cleaning were conducted using R studio packages tidyverse, stringr, dplyr, and googlesheets4.

Random Forest Modeling Pre-Processing & Analysis
To determine which factors were significantly associated with the ordinally-scaled primary outcome of repurposing project stage, we employed both Spearman Rank Correlation testing as well as random forest (RF) modeling. For this analysis, we used only survey responders that had drug-disease pairs with both (1) reported repurposing project outcomes and (2) repurposing projects where RDNPs did not deny direct involvement/support ($n_{final} = 90$). We divided RDNP leader survey questions into 2 categories: organization-specific or drug-disease pair-specific. These questions comprised hypothetical "variables" that could potentially influence success and stage of a repurposing project.

All responses were converted to a scale from 0 to 1. In the case of binary "Yes/No" questions, "Yes" and "No" responses were converted to 1s and 0s, respectively. Questions with numerical responses, such as age or staff number, were fractionated on an analogous scale from 0 to 1 (**eFile 2**). Multiple choice/list questions with many possible selections were converted to a series of binary variables. In all cases, replies of "Other" that could not otherwise be re-classified according to the "Survey Data Cleaning" **Supplemental Methods** section and could not be assumed to be "No"/0 were excluded from the model. A summary of all the variables mapped from survey questions to each model – with their associated data pre-processing steps – is included in **eFile 2**. In the RF model, outcomes were ordinally classified, with "Unsuccessful", "Early Stage without Reaching a Clinical Endpoint", "Clinical Stage without Reaching a Clinical Endpoint", "Late Stage without Reaching a Clinical Endpoint", and "Successful" being designated values of 0-4, respectively. Each variable was also independently tested for a significant correlation with the ordinally-scaled outcome of repurposing "success" using a Spearman rank-order correlation, as it is ideal for the ordinal nature

© 2025 Nijim S et al. *JAMA Network Open*.

of the data. The p-values from this test were corrected for multiple hypothesis testing (within their respective “drug-disease pair-specific” and “organization-specific” variable groups) using a Benjamini-Hochberg procedure.

Given the inherent collinearity and non-normality of these variables, we employed an RF algorithm for data interrogation and used 5-fold cross-validation for model training and accuracy assessment. We assessed accuracy of the model using an ordinal accuracy metric that employed a confusion matrix to account for the closeness of predictions to actual categories, applying diminishing weights to errors based on their distance from the correct category. Model accuracy was approximately 80% for both drug-disease pair-specific and organization-specific random forest models. We varied the ‘mtry’ parameter – the number of variables randomly sampled as candidates at each split – from 3 to 8 and used the ‘mtry’ for each model that provided the highest model accuracy. ‘mtry’ values of 5 and 7 were used for the drug-disease pair-specific and organization-specific random forest models, respectively. We set the number of trees in the forest to 10,000 to enhance the stability and accuracy of model predictions. The Gini Importance value – a measure of feature importance – for each variable in the RF model was averaged across all 5 training/testing data splits of the 5-fold cross validation.

After pre-processing as described above, we were left with 62 organization-specific and 28 drug-specific variables to test against the ordinally-scaled drug success outcomes. For each RF model, organization- or drug-specific, we eliminated responders with 1 or more non-responses to the chosen questions leaving 83 responders for the organization-specific model and 85 responders for the drug-specific model.

Other Significance Testing

Beyond RF modeling, we orthogonally tested variables against binary “success” of the reported repurposing projects (“successful” or “not successful”, according to the definition in the **Methods** section). For binary variables, Fisher’s exact and Chi-squared tests were used to evaluate categorical variables with a cell-count below and above 2, respectively; t-test and Mann-Whitney significance tests were used for parametric and non-parametric continuous variable types, respectively.

Interview Selection

From the 147 responding RDNPs, we developed a typology based on various internal characteristics and drug repurposing experience reported by RDNPs to select RDNPs. These typology characteristics are included in the deidentified image below. We selected all 15 rare disease nonprofit organizations that reported at least one subjective success endpoint to have been met. We also selected 17 organizations where no subjective success endpoints had been met yet. We selected the latter to include a spectrum of repurposing projects at diverse stages and included RDNPs with comparable typology characteristics (age, funding level, staff size, etc.) to the cohort with a successful endpoint.

© 2025 Nijim S et al. *JAMA Network Open*.

RDNP	success outcome achieved yes/no	Q19 How many full time staff members do you currently have?	Q18 What year was the organization founded?	Q23 What is the average annual funding that your organization has had in the past 3 years (both raised internally and received from external grants and philanthropists)?	Q28_3 Does your rare disease of focus currently have - Diagnostic Criteria	Q26_7 Does your organization currently have - Scientific or medical advisory board	Q28_7 Does your rare disease of focus currently have - Animal models	Q35 Have any other drugs (drugs which have not been FDA approved for your rare disease of focus), been identified as promising for your rare disease of interest?	Q42 Does your organization systematically track off-label drug use in your patient population?	Q28_6 Does your rare disease of focus currently have - Identified genetic mutation	Q26_8 Does your organization currently have - Patient registry (i.e. database where patients or	Q26_9 Does your organization currently have- Natural history study (i.e. research study	Q26_10 Does your organization currently have - Patient reported outcomes (PRO) assessment	Q26_11 Does your organization currently have - Biobank to collect patient samples (e.g. blood,	Q26_12 Does your organization currently have - Research strategy or research agenda	Q28_2 Does your rare disease of focus currently have - Treatment guidelines / Standard of Care	Q28_4 Does your rare disease of focus currently have a specific ICD-10 code	Q28_8 Does your rare disease of focus currently have - Cell lines	Q28_1 Does your rare disease of focus currently have - Clear understanding of etiology or disease	Q28_5 Does your rare disease of focus currently have - Predictive biomarkers (i.e. to help to predict
[redacted]		1	2010	Between $100,000 and $500,000	Yes	No	No	Yes	No	Yes	No	No	Yes	No	No	Yes	Yes	No	Yes	Yes
		0	2005	Between $500,000 and $1,000,000	Yes	Yes	Yes	No	Yes	Yes	Yes	No	No	No	Yes	No	No	No	Yes	No
	Yes	0	2017	Between $5,000 and $10,000	Yes	No	Yes	Yes	No	Yes	No	No	No	No	No	No	No	No	Yes	No
	Yes	0	2011	Less than $5,000	Yes	Yes	Yes	Yes	No	Yes	No	Yes	No	No	No	Yes	Yes	No	Yes	No
		2	2007	Between $500,000 and $1,000,000	Yes	Yes	Yes	Yes	No	Yes	Yes	Yes	No		Yes	Yes	Yes	Yes	Yes	No
		10	2005	Between $500,000 and $1,000,000	Yes	Yes	Yes	Yes	No	Yes	No	No	No			Yes	No	No	No	Yes
		3	1991	More than $5,000,000	Yes	Yes	Yes	No	No	Yes	Yes	Yes	Yes	Yes	Yes	Yes	Yes	Yes	Yes	No
		1	2008	Between $500,000 and $1,000,000		Yes					No	No	No		Yes					
		1	2018	Between $100,000 and $500,000	No	Yes	Yes	No	No	Yes	Yes	No	No	Yes	No	No	No	Yes	No	No
		0	2006	Between $50,000 and $100,000	No	Yes	Yes	Yes	No	Yes	No	No	No	No	Yes	No	Yes	No	No	No
		4	2000	Between $1,000,000 and $2,000,000	Yes	Yes	Yes	Yes		Yes	Yes	No	No	Yes	Yes	Yes	Yes	Yes	Yes	Yes
		0	2007	Between $100,000 and $500,000	Yes	Yes			No		Yes	Yes			Yes	Yes	Yes		Yes	
		3	2008	Between $500,000 and $1,000,000	No	Yes	Yes	Yes	No	Yes					Yes	No	No	Yes	No	No
		0	2017	Between $100,000 and $500,000	Yes	Yes	Yes	No	No	Yes	Yes	Yes	No	No	No	No	No	Yes	No	No
		1	2018	Between $10,000 and $50,000	Yes	Yes	Yes	No	No	Yes	Yes	Yes	No	Yes	Yes	Yes	No	Yes	No	No
		0	2020	Between $100,000 and $500,000	No	No	Yes	Yes	No	Yes	Yes	Yes	No	Yes	No	No	No	No	No	No
	Yes	0	2003	Between $1,000,000 and $2,000,000	Yes	Yes	Yes	Yes	No	No	No	No	No	No	No	No	Yes		No	Yes
		1	1999	Between $100,000 and $500,000	Yes	Yes	Yes	Yes	No	Yes	Yes	Yes	No	Yes	Yes	Yes	No	Yes	Yes	No
	Yes	2	2012	Between $500,000 and $1,000,000	Yes	Yes	Yes	Yes	Yes	No	Yes	Yes	Yes	Yes	Yes	Yes	Yes	Yes	No	No
		0	2014	Between $100,000 and $500,000	Yes	Yes	Yes	Yes	No	Yes	Yes	Yes	Yes	Yes	Yes	Yes	Yes	Yes	Yes	Yes
		0	2015	Between $100,000 and $500,000	Yes	Yes	No	Yes	No	Yes	Yes	Yes	Yes	Yes	No	Yes	Yes	Yes	No	No
		1	2018	Between $50,000 and $100,000	Yes	Yes	Yes	No	No	Yes	Yes	Yes	No	Yes	Yes	No	No	Yes	No	No
		0	2009	Between $50,000 and $100,000	Yes	Yes	Yes	No	No	Yes	No	Yes	No	No	No	No	No	Yes	Yes	Yes

We compared our selected organizations both within the interview group (15 vs 17 organizations) and to the total 147. We also compared those selected for interviews vs. those not selected (32 vs 115). Overall, the percentage distribution comparing these groups is included below:

		15 "met a success endpoint"	17 in "process/unsuccessful"	147 all
Funding	Less than $5,000	6.67%	0.00%	4.17%
	Between $5,000 and $10,000	6.67%	0.00%	4.86%
	Between $10,000 and $50,000	6.67%	6.25%	17.36%
	Between $50,000 and $100,000	0.00%	6.25%	10.42%
	Between $100,000 and $500,000	33.33%	50.00%	32.64%
	Between $500,000 and $1,000,000	13.33%	12.50%	13.19%
	Between $1,000,000 and $2,000,000	20.00%	12.50%	8.33%
	Between $2,000,000 and $5,000,000	6.67%	6.25%	4.17%
	More than $5,000,000	6.67%	6.25%	4.17%
Age	pre 2000	46.67%	12.50%	21.77%
	post 2000	53.33%	87.50%	76.87%
Size of staff	0 staff	40.00%	18.75%	35.62%
	1 staff	0.00%	56.25%	26.71%
	2-5 staff	26.67%	12.50%	21.92%
	5-10 staff	26.67%	12.50%	10.27%
	>10 staff	6.67%	0.00%	4.11%
Yes for Diagnostic Criteria		100.00%	93.75%	82.86%
Yes for SAB/MAB		93.33%	100.00%	92.31%
Yes for Animal models		93.33%	87.50%	78.20%
Yes for Off label drugs identified -		86.67%	76.92%	66.39%
No - do not systematically track off label use		60.00%	87.50%	87.77%
Yes for identified genetic mutation		73.33%	93.75%	82.35%
Yes for Patient registry		53.33%	75.00%	59.86%
Yes for natural history study		53.33%	75.00%	42.14%
Yes for Patient reported outcomes		42.86%	43.75%	24.82%
Yes for biobank		40.00%	56.25%	34.09%
Yes for research agenda		66.67%	87.50%	55.07%
Yes for treatment guidelines		57.14%	62.50%	51.45%
Yes for ICD code		71.43%	43.75%	47.45%
Yes for Cell lines		71.43%	93.75%	63.57%
Yes for Clear understanding of etiology or disease pathogenesis		46.67%	68.75%	49.28%
Yes for Predictive biomarkers		28.57%	25.00%	25.56%
Yes for FDA approved drugs		33.33%	12.5	29.50%

		n = 147	n = 25	n = 122	n = 147	n = 25	n = 122
	Category	# all	# interviewed	# not interviewed	% all	% interviewed	% not interviewed
funding	Less than $5,000	6	1	5	4.08	4	4.1
	Between $5,000 and $10,000	7	1	6	4.76	4	4.92
	Between $10,000 and $50,000	25	2	23	17.01	8	18.85
	Between $50,000 and $100,000	15	0	15	10.2	0	12.3
	Between $100,000 and $500,000	48	10	38	32.65	40	31.15
	Between $500,000 and $1,000,000	19	2	17	12.93	8	13.93
	Between $1,000,000 and $2,000,000	12	6	6	8.16	24	4.92
	Between $2,000,000 and $5,000,000	6	2	4	4.08	8	3.28
	More than $5,000,000	6	1	5	4.08	4	4.1
age	2000 and prior	36	6	30	24.49	24	24.59
	post 2000	111	19	92	75.51	76	75.41
staff size	zero	52	6	46	35.37	24	37.7
	1 to 1.5	40	7	33	27.21	28	27.05
	2 to 5	32	6	26	21.77	24	21.31
	6 to 10	15	5	10	10.2	20	8.2
	11 or more	7	1	6	4.76	4	4.92
etc	Yes for Diagnostic Criteria	123	24	99	83.67	96	81.15
	Yes for SAB/MAB	136	24	112	92.52	96	91.8
	Yes for Animal models	118	23	95	80.27	92	77.87
	Yes for Off label drugs identified -	107	22	85	72.9	88	69.67
	No - do not systematically track off label	130	18	112	88.44	72	91.8
	Yes for identified genetic mutation	123	20	103	83.67	80	84.43
	Yes for Patient registry	90	14	76	61.22	56	62.3
	Yes for natural history study	66	15	51	44.9	60	41.8
	Yes for Patient reported outcomes	44	8	36	29.93	32	29.51
	Yes for biobank	60	10	50	40.82	40	40.98
	Yes for research agenda	85	19	66	57.82	76	54.1
	Yes for treatment guidelines	80	15	65	54.42	60	53.28
	Yes for ICD code	75	15	60	51.02	60	49.18
	Yes for Cell lines	100	21	79	68.03	84	64.75
	Yes for Clear understanding of etiology o	77	14	63	52.38	56	51.64
	Yes for Predictive biomarkers	48	6	42	32.65	24	34.43
	Yes for FDA approved drugs	49	6	43	33.33	24	35.25

© 2025 Nijim S et al. *JAMA Network Open*.

eFigure 1. Distribution of Organizational Demographics of ROADMAP Survey Respondents Compared With the Broader US-Based RDNP Landscape

The distribution of several RDNP features was extracted and evaluated to compare ROADMAP RDNPs with the broader pool of non-responding RDNPs meeting inclusion criteria. Differences in distribution of (**a**) organizational age, (**c**) annual number of compensated employees, (**d**) geographic distribution, and (**e**) number of rare diseases of focus were not statistically significant. ROADMAP RDNPs, however, had a significantly higher median total revenue ($355,390) compared to non-ROADMAP RDNPs ($172,393.50; p-value < 0.0001; Kolmogorov-Smirnov). Of note, for distributions and analyses related to total revenue (**b**) and compensated employee number (**c**), 1 RDNP in the “ROADMAP” cohort and 5 RDNPs in the “Non-ROADMAP” cohort were indeterminate and counts for these analyses were adjusted accordingly (n = 546 and 146, respectively).

© 2025 Nijim S et al. *JAMA Network Open*.

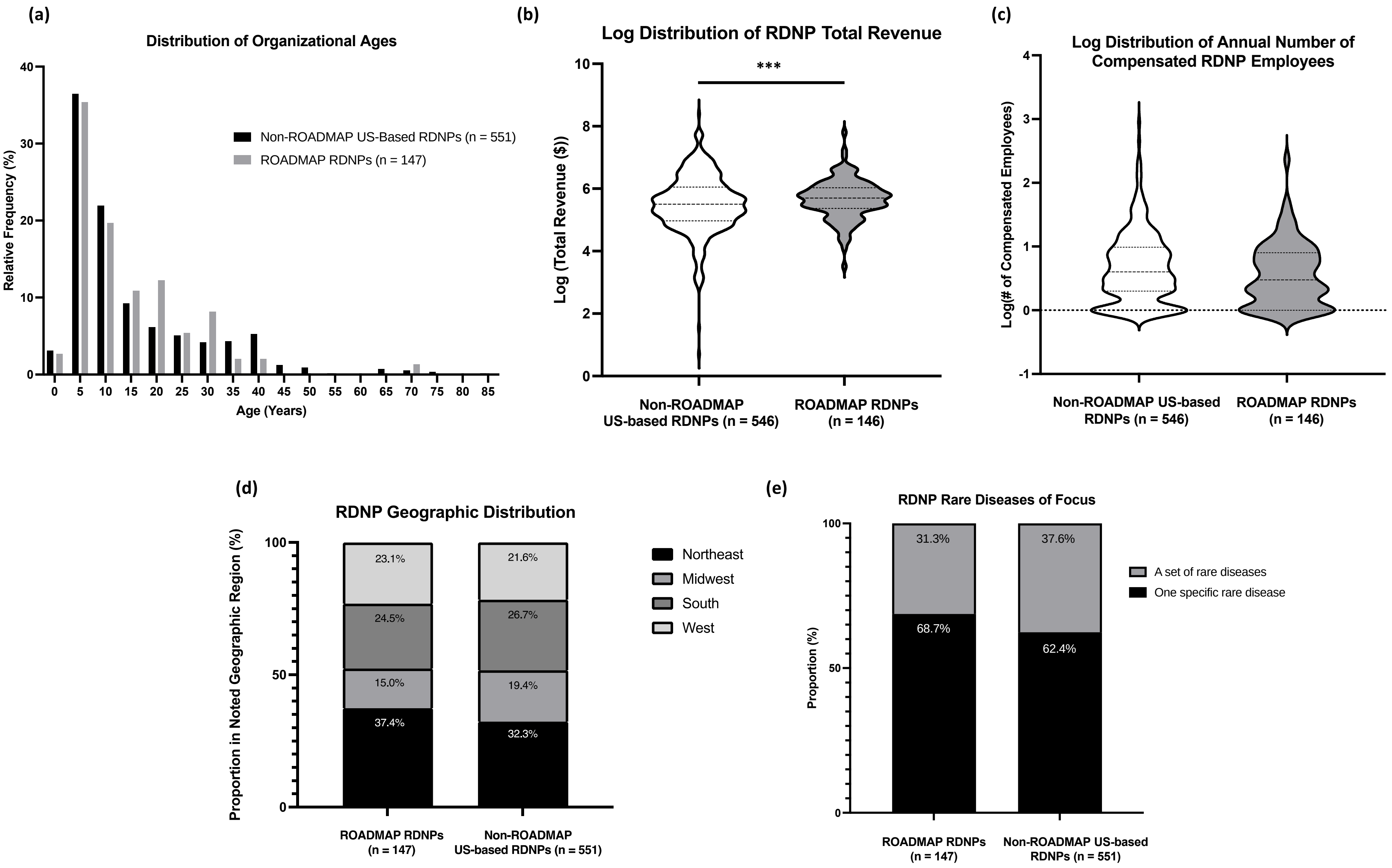

© 2025 Nijim S et al. *JAMA Network Open*.

eTable 3. Drug-Disease Pair-Specific and Organization-Specific Variables Associated With Successful Repurposing Project Outcomes

We used three orthogonal analyses to predict variables associated with “successful” repurposing outcomes: (1) two random forest (RF) models of drug-disease pair-specific and organization-specific variables to predict repurposing project status (ordinal primary outcome); (2) ordinal outcome significance testing (Spearman correlation coefficient); and (3) binary outcome (“successful” vs. not “successful”) significance testing (variable class-specific: “drug-disease pair-specific” and “organization-specific”). Seven variables were of high Gini importance in the two RF models and had statistically significant associations with ordinal project outcome on Spearman rank-order correlation testing. When the primary outcome was binarized, 5 of the 7 variables were of nominal significance.

Factor	Variable Type	Ordinal Outcome RF Model (n = 83 for Organization-Specific Variables, n = 85 for Drug-Disease Pair-Specific Variables)			Binary Outcome Statistical Analysis (nmax = 34)		
		Gini Importance	*Correlation*	*Adjusted P-Value (Spearman)*	*Non-Adjusted P-Value*	*Adjusted P-Value*	*Test*
* Organization Supports Drug-Disease Pair through Patient Recruitment into Clinical Trials	**Drug-Disease Pair-Specific**	3.90	**+ 0.50**	**0.00005**	**0.036**	>0.05	Fisher's Exact
* Number of Research Methods Used by Organization to Identify Drugs	**Organization-Specific**	1.51	**+ 0.40**	**0.003**	**0.004**	0.09	t-Test
* Patient Education is in Top 3 Activities of Focus	**Organization-Specific**	1.05	**+ 0.36**	**0.01**	**0.03**	>0.05	Fisher's Exact
* Reported Number of Promising Drugs Identified for Rare Disease of Focus	**Organization-Specific**	1.76	**+ 0.42**	**0.003**	**0.002**	0.09	Mann-Whitney
* Organization Fundraises Using a Family Foundation	**Organization-Specific**	0.78	**+ 0.36**	**0.01**	0.06	>0.05	Fisher's Exact
* Organization Fundraises Using Employer Matching	**Organization-Specific**	1.78	**+ 0.47**	**0.0005**	>0.05	>0.05	Chi-squared
* Organization Supports Researchers through Non-Financial Research Support	**Organization-Specific**	0.69	**+ 0.33**	**0.02**	**0.02**	0.31	Fisher's Exact

© 2025 Nijim S et al. *JAMA Network Open*.

eFigure 2. Random Forest Models of Drug-Disease Pair-Specific and Organization-Specific Variables Associated With Successful Repurposing Project Outcomes

We used three orthogonal analyses to predict variables associated with “successful” repurposing outcomes: (1) two random forest (RF) models of drug-disease pair-specific and organization-specific variables to predict repurposing project status (ordinal primary outcome); (2) ordinal outcome significance testing (Spearman correlation coefficient); and (3) binary outcome (“successful” vs. not “successful”) significance testing (variable class-specific: “drug-disease pair-specific” and “organization-specific”). We include model outputs from two RF models of (**a**) drug-disease pair-specific variables and (**b**) organization-specific variables from the ROADMAP survey. Repurposing project stage is ordered ordinally (in darker shades of purple on the horizontal bar) from 0-4 (0 = abandoned/“unsuccessful”; 1 = “early stage without success/abandonment”; 2 = “clinical stage without success/abandonment”; 3 = “late stage without success/abandonment”; 4 = “successful”). The horizontal “success” bar beneath (in green) denotes binary “success”. The gradient bar under each heat map denotes a spectrum of values from 0-1. If the variable is categorical (0 vs. 1), “0” is denoted by the beige-yellow color, whereas “1” is indicated in dark red-brown. Continuous variables (e.g., “number of drug-specific drug research methods used to identify a drug-disease pair”) have values, and shades, in between.

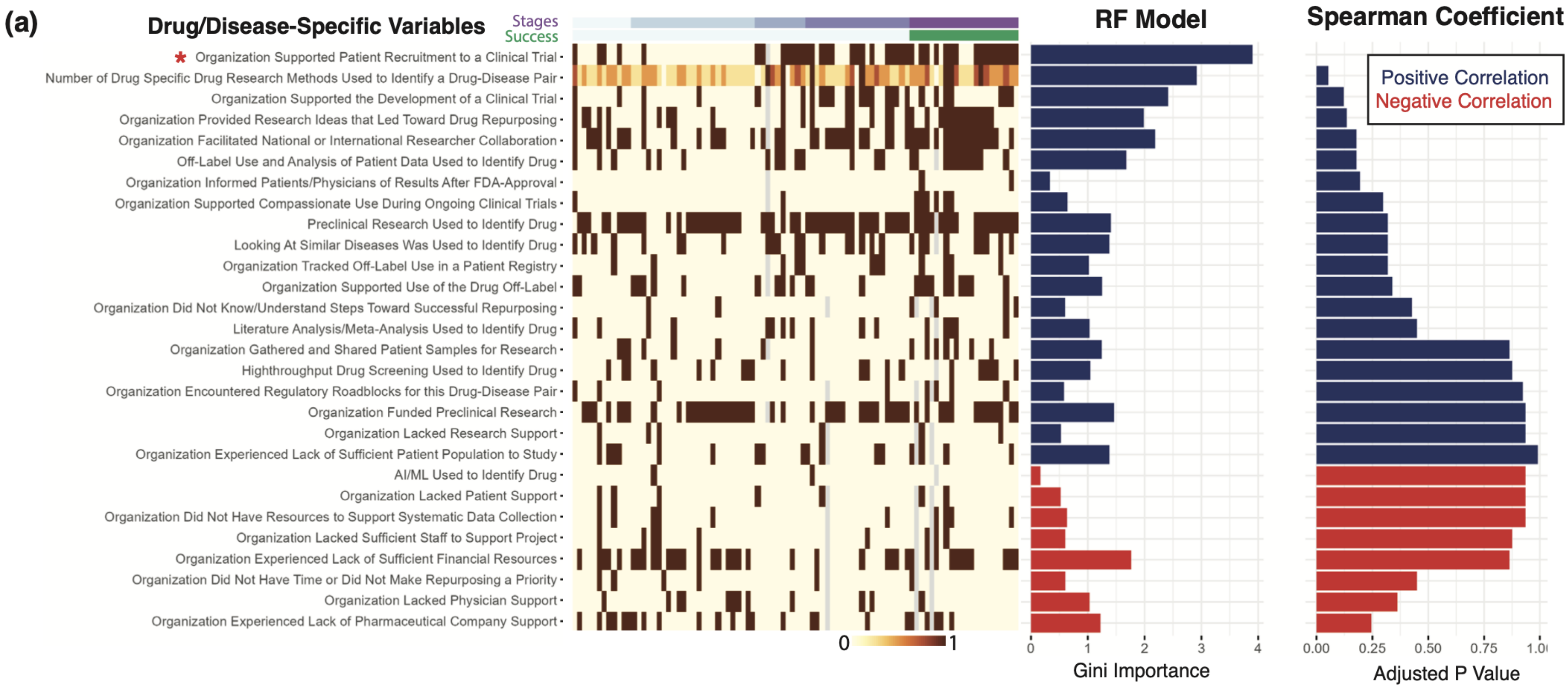

© 2025 Nijim S et al. *JAMA Network Open*.

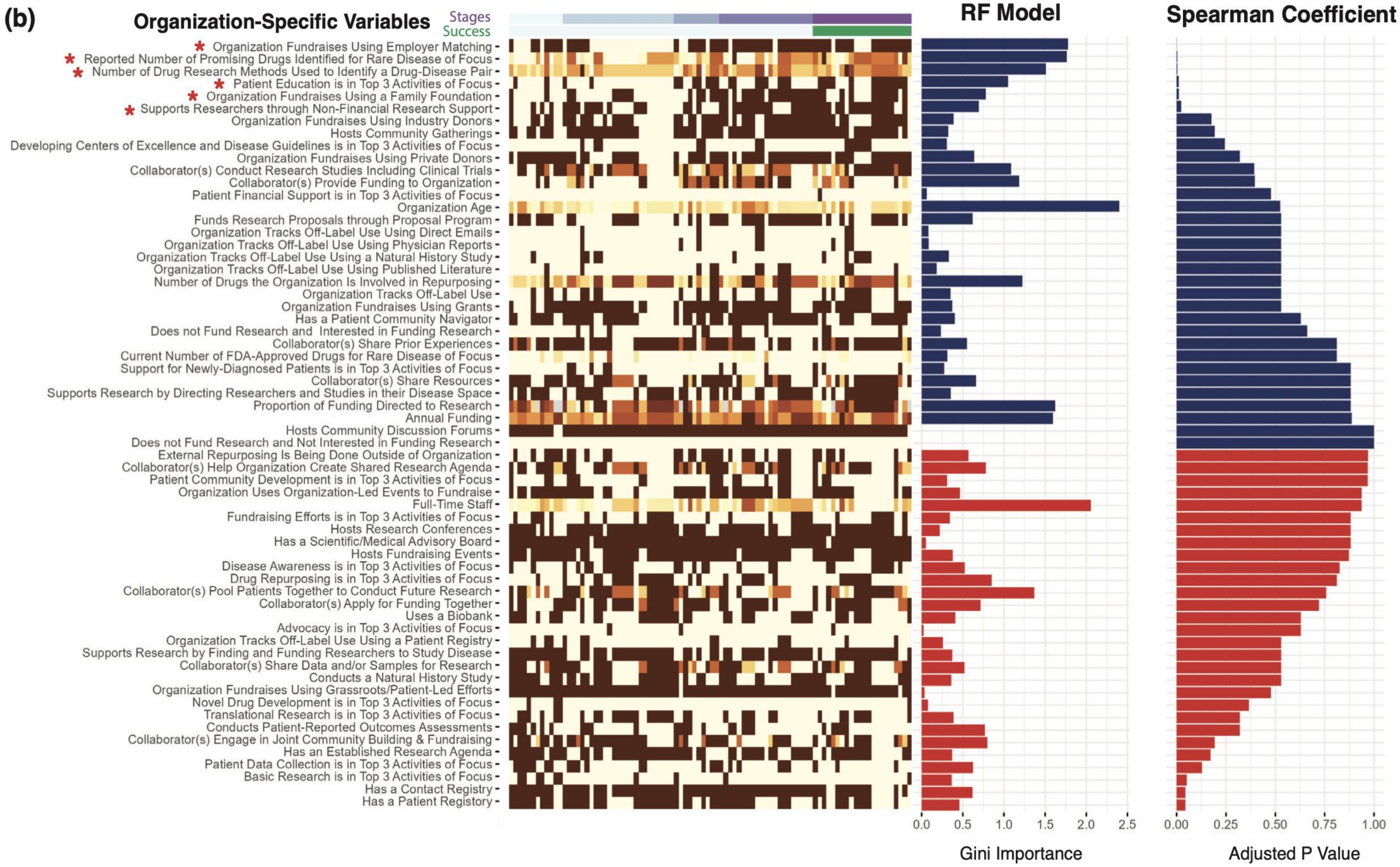

© 2025 Nijim S et al. *JAMA Network Open*.

eTable 4. Themes, Subthemes, and Illuminating Quotations From ROADMAP Rare Disease Nonprofits

Following interviews with leaders of 25 RDNPs, 5 chronological themes were consistent with narratives of their repurposing experiences: (1) enabling conditions for drug repurposing, (2) identifying a promising drug candidate, (3) validating a drug candidate, (4) clinical use/testing, and (5) reaching an optimal endpoint. Two other non-chronological themes were discussed during interviews: (6) roadblocks and challenges and (7) opportunities/recommendations from RDNPs. Each theme includes multiple sub-themes, and illuminating quotes are listed below. Boxes are colored green, yellow, and red according to the outcome of the project (e.g., green: met a success outcome of FDA-approval or off-label use with subjective clinical benefit; red: abandoned/not successful; yellow: has not reached a significant afore-defined clinical endpoint of either success or abandonment).

**eTable 4. Themes, sub-themes, and illustrative quotes from RDNP representatives**		
**Themes**	**Sub-Themes**	**Quotes**
**1. Enabling drug repurposing**	a. Funding support	"However, the organization... was founded about 10 years ago, when there was a need to gather some funding for a neurologist that was at the NIH... to basically... fork the trial and then create one specifically for [***Disease***]. So, the organization was very involved in the beginning... in primarily a funding way." (***RDNP 1; Clinically-Beneficial Off-Label Use***)<br><br>"Well, you know, as a nonprofit organization... we don't have steady income from anywhere, so we have to go out and raise the funds. So, we have focused most of our efforts on joint fundraising programs to support the program that was taking place in [***European country***] and was successful... the drug was approved in Europe. And, so, that sort of helped to lay the groundwork for us, where they had a population that was easily accessible." ***(RDNP 2; Clinically-Beneficial Off-Label Use***)<br><br>"In 1994, 1995, growing from… identifying 10 patients with this very rare disease… [to] now 3600 in the database… it started in a church basement, where you have a handful of scientists and a handful of patients starting to talk about how this could work, now how do we collaborate to raise money to fund research?" (***RDNP 16; FDA-Approved***)
		"Maybe [***#***] years ago… there was a trial planned, a multicenter pediatric trial with leaders in the space… And they were putting together a proposal...at the last minute, they determined that it was just going to be way too expensive, and they dropped it. And since then a natural history study that came out of that work... it has been funded by the NIH. So, there is interest from the researchers to do it in the right way... it just takes money." (***RDNP 20; Clinical Stage without Clinically-Significant Endpoint***)

© 2025 Nijim S et al. *JAMA Network Open*.

		“For us, it’s impossible to cover…we need partners, we need bigger donors…at the moment, it’s impossible…The foundation contracted a new employee a month ago…to try to get big donors, not the usual donors…We are funding that work [high throughput screening], and again, it is seed funding, a small grant of $50,000…We have some experts on our field…they say they can synthesize the drug and keep going” (***RDNP 9; Abandoned***)<br><br>“We needed money. We needed about $250,000… to run a cohort in the United States… The results were not what they were anticipating, and then COVID hit, so everything stopped” (***RDNP 25; Abandoned***)
	b. Research support	“[We were] able to actually take compounds [proposed by external collaborators] in house and give some preclinical data [because we already set up and streamlined a] therapeutics core at [***academic institution***]…This was really important… to really standardize and put infrastructure in place to be able to test… both internal compounds and also compounds or drugs from external collaborators, where researchers from other institutions… could also collaborate… [and fill research] gaps” ***(RDNP 19; Clinically-Beneficial Off-Label Use)***<br><br>"So, I think the research was the biggest component for making a decision to move forward, because the physicians were there, they were studying and analyzing people over periods of time, both in [***European country***] and in the United States and gathering data, sharing data. And… a key thing is there's…a [research] meeting every year in Europe, that we all get together, patients and physicians are invited…and people from all over the world will come and share their information. And… that is one of the situations that is a catalyst… for deciding what's the next step... Do we have enough information to go [to] the next step, and… what's the best way to do that. And… all the important players around the world are there and can give their input. That makes it easier in some ways to make a decision and move forward..." (***RDNP 2; Clinically-Beneficial Off-Label Use***)<br><br>“Our community [supported research by] really encouraging people to give samples. So, at the time of the publication… [the research team we were working with] started to follow that up with human screening.” (***RDNP 23; Clinically-Beneficial Off-Label Use***)<br><br>“The foundation facilitates research by giving out grants to investigators [of interest], and from the start, there has been a deep interest in identifying complementary ways to target pathways” (***RDNP 16; FDA-Approved***)
		“The biggest thing is to set up centralized research capabilities, so that you don’t have to continually find academics to recreate the same capabilities over and over…We needed to create models and model repositories and a biobank for many different purposes…none of this would have been possible until cell lines existed.” (***RDNP 6; Clinical Stage without Clinically-Significant Endpoint***)
		“I started the nonprofit in [***year***]... From there, we started spreading awareness, fundraising… and we started investing in getting a really solid understanding of the mechanisms of disease. We invested in a repository of patient samples. So, obviously, we started with my [family member], but we collected samples from a few other families, with children affected.” (***RDNP 14; Abandoned***)

© 2025 Nijim S et al. *JAMA Network Open*.

	c. Partnering & collaboration	“We have an initiative called the [***Name***] Initiative, which is a collaboration between the [***Name***] Institute, [***Second Non-Profit Name***], and [***our RDNP***]… We brought together experts from all over the world… people that were doing cell models, animal models, transcriptomic, proteomic [analyses]…Before I came to the foundation, [we also had] an initiative called the [***Disease***] Preclinical Consortium, which was a group of labs we [work with to] bring drugs to the clinic that have shown value in a preclinical model. With [a group of] about five labs, who would work together [to] select certain drugs, they [would] test them in their preclinical model and bring them to the clinic. So that was the first attempt to look at drugs for [disease] repositioning, repurposing, or [drugs anywhere] in clinical development… Then those drugs were brought to the [***clinical trials group name***]” (***RDNP 5; FDA-Approved***)<br><br>“We’re connected with academics… We know who’s working in our space and connect with them globally. We also assist in collecting patient data for their studies” (***RDNP 15; FDA-Approved***)<br><br>“We collaborated with [***pharmaceutical company***] and used data from our registry to help with EMA approval… Our foundation’s funded project provided the necessary longitudinal data” (***RDNP 16; FDA-Approved***)
		“We were fortunate to have a partner with an academic institution and [***another organization***] … They built the yeast models based on existing research, and we donated to support the project” (***RDNP 17; Early Stage without Clinically-Significant Endpoint***)
		"I think one thing I wish I had done was get more medical professionals involved in our decision to help recruit. I think I just figured you know, it was IND-approved… Afterwards, I was like, I wonder… we don't have any MDs on our advisory board. So, there was nobody easy to ask. But I kind of wish [we] had maybe just reached out *ad hoc* to some people to say, you know, do you think it's a good idea." (***RDNP 8; Abandoned***)<br><br>“It is detrimental… to have two organizations. Because if it’s not collaborative, we’re going to lose. All of us are going to lose… Splitting the patient community… all these organizations trying to do everything at once… You can’t. You’ve got to focus on… you have to prioritize.” (***RDNP 25; Abandoned***)<br><br>“We now require people to work together, and we require them to share… [to] make it very publicly known when they’re not sharing” (***RDNP 12; Abandoned***)

© 2025 Nijim S et al. *JAMA Network Open*.

	<u>d. Patient support</u>	"[During repurposing], [we were also] preparing the patient community to be ready if, in fact, we were going to be called to clinical trials… We continued to engage the [***disease***] community in… safety trials. There is a management aspect of expectations on that… [During ***clinical trial name***], we paid for patient travel and… supported the patients. We distributed all the payments to the site, so [***our RDNP***] actually found ways to save money by becoming the payment administrator to the site's recruited patients. [We] paid traveled costs and then we ended up funding the last year of monitoring in [***foreign country***] when funds ran out. So, this was, year to year, month to month… finding more money collaboratively across industry, patient organizations, government to keep this trial going." (***RDNP 16; FDA-Approved***)<br><br>"What we do as an organization… we help [patients], direct them for care… [For example, for repurposing coverage] our doctors are unbelievable. We've reached appeal letters, I've written appeal letters. And more often than not, it's covered… We supported compassionate use… We provided a lot of education, a lot of webinars. We gave it at our patient conferences…we really educated the community… Now there's a population of patients [for which the repurposed drug] doesn't work at all, or it works and then it stops. So, our physicians get together pretty frequently… And there's a lot of dialogue and quarterly meetings" (***RDNP 11; Clinically-Beneficial Off-Label Use***)
		"We have another patient in [***country name***], [our RDNP] pays to have, for her to have that [repurposed drug] because the [***country name's***] insurance only covered it for like two months" (***RDNP 13; Early Stage without Clinically-Significant Endpoint***)
		"We're trying to be that support system for our patient community as well. We've been collecting information and guiding families. For example, we worked with the manufacturer and my [***family member's***] geneticist to get compassionate use approval for a drug [***repurposed drug name***] that had preclinical data. While the trial didn't lead to significant improvements, it was important for us to be involved in ensuring families had access to potential treatments and understood the options available. We know once we share any new information, families rush to figure out how to access it for their child. So, we need to provide clear guidance on dosing, side effects, and how to obtain the drug" (***RDNP 14; Abandoned***)
**2. Identifying a promising drug candidate**	<u>a. Targeting mechanisms related to disease</u>	"[In our case], the search for a drug didn't start with the intent that it has to be a repurposed drug…[it] started in the context of… let's understand the molecular pathogenesis. And based on that, we can see which targets seem right… And, as it so transpired, when the discovery of the genetic mutation and the main pathway that mutation was targeting, there was a ready-made drug that already existed…The first breakthrough in [***our disease***] really happened when it was discovered that mutations in the [***identifying class of genes***] genes caused [***our disease***]. And the next step actually happened… in the fruit fly experiment, where it was discovered that these mutations… regulate the central [***name of pathway targeted by repurposed drug***] pathway that leads to [***our disease's effect***]." (***RDNP 16; FDA-Approved***)<br><br>"We needed to repurpose a drug. The focus was to find a drug, so we followed the science. We looked at the [***pathway name***] pathway and said, 'Okay, is there a drug?' That's what guided us, letting the biology lead the way" (***RDNP 3; Clinically-Beneficial Off-Label Use***)

© 2025 Nijim S et al. *JAMA Network Open*.

		“[The possibility of using this repurposed drug] came from… three different groups [who] all independently found that the target of [***the repurposed drug***], which is [***drug target***], was very active in [***disease***] and that [***the repurposed drug class***] were very efficacious in [***disease***] cells… All of [***drug class inhibitors***] turned up among the most active compound classes… It was very obvious that it was a low-hanging fruit for repurposing” (***RDNP 6; Clinical Stage without Clinically-Significant Endpoint***)
	b. Drug-centered screening approaches	“In [***year***], [we had] a large collaborative consortium where we brought together experts from all over the world…[so everyone] came together [who] were doing cell models, animal models, transcriptomic [and] proteomic [analyses]….They tested [***repurposed drug***] in the cell models and they tested it in the animal models, and it was incredibly powerful… [But] nobody understood what was going on. They had to do [the] transcriptomic and proteomic studies to actually figure out that this molecule was of course hitting [its mechanism], but also hitting other mechanisms that were super relevant for [***our disease***] too… This drug apparently had value across all models. So [it was] a molecule that we [could not] ignore, even if it was [already] approved for a completely different mechanism” (***RDNP 5; Clinical Stage without Clinically-Significant Endpoint***)<br><br>"[After the previous project], we initiated a new zebrafish grant… [with] the hope that it could be used for drug screening. So, [using] a library of FDA-approved drugs, [we are] looking for rescue of a muscle deficit in those fish [with] a private company... We are funding a characterization of the model and [the testing of] several drugs on it... any drug that's been identified in the literature as potentially beneficial to [***disease name***]" (***RDNP 8; New Efforts: Early Stage without Clinically-Significant Endpoint***)
		“One of the main priorities really, from the beginning, was building more predictive drug screening models for [***disease***]. So [***disease***] impacts [***identifying cell type***] cells called [***identifying cell type***]…Once we figured out how to [model] this [through stem cell models]… it did enable large-scale drug screens to try to see what rescues a [***identifying cell type***] with [***disease***] in a laboratory setting… With our partners at other institutions, like 10 or so years ago, we started screening 1000s of compounds…big compound libraries that you could buy…And we realized through that drug screen there were FDA-approved drugs, in development for other indications, and supplements that seemed to be somewhat effective in rescuing [***cell type***] in a dish in the lab… but one of the major issues [was] the issue of penetration” (***RDNP 19; Abandoned/Unsuccessful***)
	c. Data from initial off-label/human use	“[***Repurposed drug***] was identified by a pediatric doctor who was treating a [***disease***] patient for [***another disease name***] [for which] [***repurposed drug***] is the standard treatment. And he noticed that [markers of disease]… seemed to improve when he went off it. After the [***other disease***] cleared up, everything got worse [for this disease]. So, he put him back on it and things got better again. He published this story and… a series of case studies… and started putting his patients on it… So, it's available as an off label drug, [and] since then, people have been using it… More than 60% of patients in [our] surveys are taking it... and it's higher in Europe, because it's much more widely accepted… [But] there was great controversy [regarding whether] it was going to affect [if] clinical trials would ever be done, because people were starting to use it in these case studies. So, there is a little bit of evidence published…mostly observational [and] there were two small clinical trials, one of which was funded by [***our RDNP***]…" (***RDNP 20; Clinically-Beneficial Off-Label Use***)

© 2025 Nijim S et al. *JAMA Network Open*.

		"[***Research institute name***] is doing some basic/translational science on that, [but] we kind of worked in reverse order. We took the real-world data from our population and then returned that information when we observed the changes in our children after taking [***drug name***]. Using the real-world data is when we discovered the sensory processing mechanism that [***disease gene***] controls in the frontal somatic cortex of the brain, which is [pathologically] depressed. [We put] two and two [put] together [from there] because [***repurposed drug name***] also has an indication [in] [***another disease with similar mechanism***]." (***RDNP 25; Early Stage without Clinically-Significant Endpoint***)
	<u>d. Looking at similar diseases</u>	"An enzyme process that happens in [***our disease***] [was] very similar to [***another disease***] … [and] so then [the] idea [was to] study the parallels -- it's the same enzyme process… And it worked amazingly well… It was discovered that when patients took [***repurposed drug***], it reduced [***disease pathological substance***] by 95%... [and] that started the clinical trials, both in North America and across Europe." (***RDNP 2; Clinically-Beneficial Off-Label Use***)<br><br>"The targets are all very similar… In the case of [the ***repurposed drug***] … it eliminates [***identifying immune cell***]. It doesn't matter if it's [***our disease***] or the [***comparable disease***], you're still eliminating [***a product of the identifying immune cell***] either way. It works for both diseases, but there's evidence it works better on some than others" (***RDNP 15; FDA-Approved***)
		"Even though these diseases [RDNP's disease and other disease] manifest very differently, a lot of internal pathways like [***identifying disease mechanism***] are similar across [***identifying class***] diseases. One of the things that we saw with [***repurposed drug***] is that maybe drugs that work… for other [***identifying class***] diseases… could work for [***disease name***]… because even though it's a different [***identifying cell***] type that is impacted, they seem to respond the same, in very similar ways to certain drugs" (***RDNP 19; Abandoned***)
	<u>e. Computational approaches</u>	"We worked with the [***academic institution***] *Drosophila* lab on computational models… and they did a computational screen to identify [***repurposed drug candidate***] as a potential candidate for [***disease***]" (***RDNP 22; Early-Stage without Clinically-Significant Endpoint***)<br><br>"We had researchers in [***country***], do some screening on 3800 compounds… and they came out with [***gene class***] targeting molecules that would be the most efficacious in the cell lines and the PDX [patient-derived xenograft] models. And also, they found that in… [***disease***] models that we used to determine whether or not the drugs are toxic for [***disease***] patients, they weren't toxic" (***RDNP 12; Early-Stage without Clinically-Significant Endpoint***)
**3. Validating a drug candidate**	<u>a. *In vitro* validation</u>	"Once the central discovery of [***pathway class name***] pathway was discovered, it was relatively easy to make the connection that there is a pre-existing drug… this was very rapidly tested in a preclinical model, which showed the desired, expected results… the gene discovery and the fruit fly experiments were serendipitous things… once the linkage of the gene mutations to [***pathway name***] pathway was discovered, that first bit of preclinical science after that was funded by [***our RDNP***] and then [***the RDNP***] played a central role with funding as well as with organization of both the [***trial 1 name***] and [***trial 2 name***]." (***RDNP 16; FDA-Approved)***

© 2025 Nijim S et al. *JAMA Network Open*.

		“[We initiated] the [***RDNP name***] therapeutics core in [***year***] at [***academic institution***], and this was to fill this gap that we saw [where repurposed drug candidates and other treatments were being moved] into clinical trial with really no preclinical data around them, specifically in [***our disease***]. So, we established this in order to be able to test compounds that we find [internally] and from external collaborators. [We tried to] really standardize and put infrastructure in place to test this… This was another place [we used for ***repurposed drug***] to be able to actually take compounds in house and try to give some preclinical data that would support the program or [provide] feedback” ***(RDNP 19; Clinically-Beneficial Off-Label Use)***
		“Some of the initial rationale was published back in [***year***] by the [***foreign country***] group…[That group] also did a chemical screen and their chemical screen was done in partnership with [***pharmaceutical company***]. And that was a relationship that we had set up [at our research workshop]…and [***pharmaceutical company***] provided a number of [non-patented] compounds…And so that screen also turned up [***repurposed drug class***] as one of the most active compound classes…Another half dozen or more, maybe even, maybe eight or ten different, compound screens that were enriched for approved drugs or druggable targets turned up [***repurposed drug class***].” (***RDNP 6; Clinical Stage without Clinically-Significant Endpoint***)
	b. *In vivo* validation	“So there were two approaches that were taken…One of them was with mouse iPSCs [induced pluripotent stem cells] and the second was in worms… In worms, [the research team] was basically looking at the [***human equivalent structure***] [but] a much simpler system. And they were able to see that those aggregates were basically resolved, with the administration of the [***repurposed drug***]… [but the screen] was targeted… 20 or less…[since] they had a mechanism that they were targeting.” (***RDNP 23; Widespread Off-Label Use***)
		“Based upon the *in vitro* data that the [***research organization***] generated, we then tested two [***drug class inhibitors***]… in mouse models through the drug screening program. And indeed, they were active in [***disease***] models, one of them in particular was quite active” (***RDNP 6; Clinical Stage without Clinically-Significant Endpoint***)
		"We've been supporting research for a while… this was our first project that was related to repurposing…. [and because of early pharmaceutical company data from presentations], [***repurposed drug class candidate***] was on our radar… We had a researcher [with whom we have]... a long-term relationship... [who] applied to us for funding for some more basic research [with our] mice [models] in her lab… We introduced that investigator to a contact at [***pharmaceutical company***] to facilitate them getting that compound... [and] she completed her mouse study...[and] the results did not support moving forward... [After], [***pharmaceutical company***] did go to... human clinical trials with this compound in [***another similar disease***]. And it didn't meet its milestone [either]." (***RDNP 8; Abandoned***)
	c. Real-world evidence	"Some of our researchers discovered the [***identifying disease-specific mutation***] in [***disease***] in [***year***]. After that, there was a doctor in [***country***] who tried a [***drug class targeting that mutation***] under Managed Access, and had some success…And so he started this drug [in an end-stage patient] and [the patient] had a pretty immediate response and renal function improved, heart function improved… After that happened, he [gathered] about 18 people… [and he] published [it] in a case [series]… That was the second hit that led us to what came next…People in the US started applying for compassionate use and getting on the medication [and] there was… enough movement and action that [***pharmaceutical company*** conducted] a retrospective study of all the people who have taken the medicine via compassionate use… between pre-clinical and clinical [studies].” (***RDNP 7; FDA-Approved***)

© 2025 Nijim S et al. *JAMA Network Open*.

		"The discoveries really happened initially in the field of [***another disease***]. And what we found was that the mutations that are causing [***the other disease***] are the same mutations that are causing [***our disease***]. In fact, the first phase two studies that I'm referring to, those phase two studies actually happened in patients with [***the other disease***]. And, as it so happens, that a subset of patients with [***the other disease***] also have [***our disease***]. And when you looked at that phase two data [retrospectively], you found that the benefit was happening in patients with [***our disease***] as well. Which then led to a phase three trial, specifically for [***our disease***], but a lot of these discoveries and the earlier trials actually utilized the [***other disease***] community." (***RDNP 16; FDA-Approved***)
		"Real-world data was key. Families reported that a subpopulation of children showed improvement in behavior and sleep after using a drug that hadn't been tested for this purpose, which sparked further investigation" (***RDNP 25; Early-Stage without Clinically-Significant Endpoint***)
**4. Clinical use/testing**	a. Clinical trials	"Because it was a drug repurposing effort, they were able to move right into a large phase two, three study… the hope is that this is going to be approved on the strength of a single clinical trial, which is obviously far accelerated from a traditional drug development pathway" (***RDNP 19; Clinically-Beneficial Off-Label Use***)<br><br>"Once the central discovery of [***pathway name***] pathway was [made], it was relatively easy to make the connection that there is a pre-existing drug [targeting this] pathway… this was very rapidly tested in a preclinical model, which showed the desired, expected results… and it was then taken to single center phase two trials, one in [***city name***], and one [of] similar timeframe in [***international country***]. And they both showed very good results, which subsequently led to the design and conduction of the pivotal phase three trial, which showed the efficacy that led to the FDA and regulatory approval of [***repurposed drug***]" (***RDNP 16; FDA-Approved***)<br><br>"[The repurposed drug candidate trial was first conducted as] phase zero, phase one… it was positioned as a safety study, because it was the first time in this population" (***RDNP 23; Clinically-Beneficial Off-Label Use***)
		"We were successful in convincing [***pharmaceutical company***] to sponsor a proof-of-concept trial. And we had, I think, 8-10 of our centers at that time, lined up… three, four of the sites were activated… nine patients were seen… [and] the nine patients did amazingly well." (***RDNP 11; Late Stage without Clinically-Significant Endpoint***)
	b. Observational studies / Off-label use	"[***Pharmaceutical company name***] applied for approval [of the repurposed drug candidate] through the EMA. And the EMA granted them conditional approval, with the specification that the long-term safety and efficacy of this drug needs to be studied in a prospective manner. And that's where we, as it so happened around the same time, we already had an existing prospective longitudinal natural history registry. And we were able to collaborate with [***pharmaceutical company name***] and tell the EMA that we will use the data from this registry and give it to [***pharmaceutical company name***] to use that data to help with full EMA approval. And after four years of longitudinal data collection, we were actually able to obtain full EMA approval just a few months ago." (***RDNP 16; FDA-Approved***)

© 2025 Nijim S et al. *JAMA Network Open*.

		“[After] all of [our research] happened in mice and worms, [the research team we were working with] started to follow that up with human screening… but things quickly moved to test it in people because the side effects are really very minor for this drug.” (***RDNP 23; Clinically-Beneficial Off-Label Use***)
		“We started with off-label use, and people are just trying it on their own. We want to get the natural history study done to start documenting their progress… We’re tracking what people are taking over the counter and measuring their [identifying pathologic substance] levels” (***RDNP 24; Early Stage without Clinically-Significant Endpoint***)
**5. Reaching an outcome/optimal endpoint**	a. FDA-Approval	"Well, as far as repurposing, [FDA approval is] our primary goal. With FDA and EMA approval, there would not be [denial of] at least off-label access to the drug. Plus, with FDA approval, everything can go public, so to speak… then there's much information that we can give to patients [to say] you really need to be on [***drug name***] and here are the safety issues. It will make it much easier to educate patients and put that information out there, because it will be for [***our disease***] and they'll know exactly what's going on. And the doctors will as well." (***RDNP 2; Clinically-Beneficial Off-Label Use***)<br><br>“[Over a 4-year time period], [everyone prepared] for FDA submission and [we were] going through the submission process. [During this period], the drug went off patent, and the [pharmaceutical company] interest… was understandably not so great. So, citizens petitioned, working through a lot of just incredible, heroic efforts… everyone coming together to get this across the line for FDA approval… This [citizen] petition was to change the product label for [***drug name***] in the absence of an application from the manufacturer…but [ultimately, we were able to do so using the preferred route with the manufacturer after FDA discussions with the company]" (***RDNP 16; FDA-Approved***)
	b. Alternative endpoints and off-label use	"[Repurposing] started very early on when we saw potential drugs that seemed to be helping some people... there were a bunch of studies to document how [this drug] was working in [***disease name***] ... More than 60% of patients in [our] surveys are taking it... and it's higher in Europe, because it's much more widely accepted... Probably the most frustrating thing [about lack of formal recommendation/approval] for patients is they say ‘when I go off [***off-label drug***], I feel worse. I feel better when I go back on.’ [I think] finding evidence-based ways to document quality of life issues [is important] because that's why [***off-label repurposed drug***] seems to be used so much… It seems to really help quality of life, even though it hasn't been proven to conclusively change disease course... And is it fair to say that there has been no study [for ***our disease***] that has included quality of life measures? … The treatment guidelines are being updated right now. And they differ, depending on the society issuing them" (***RDNP 20; Clinically-Beneficial Off-Label Use***)<br><br>"It is [now] being used widely off-label. In fact, in a lot of kids… They're trying to roll people off the non-recruiting trial to commercial supply and work with their insurance companies so that they can make room for new people… I've been off-label commercially with my [***family member***] since [***year***]. Since then, there were a handful more recently. In the last year, there's dozens now." (***RDNP 1; Clinically-Beneficial Off-Label Use***)<br><br>“Over the last decade, people have been using [***repurposed drug***] off label… [For] patients, it’s been a lifesaver. Now there’s a population of patients it doesn’t work for at all, or it works and then it stops… [but] people are still using it off label. Ironically, it’s paid by Medicare and covered, and [that was done] without a phase 2/3 trial” (***RDNP 11; Clinically-Beneficial Off-Label Use***)

© 2025 Nijim S et al. *JAMA Network Open*.

		“[Right now], my thinking is, and we’ve…had some weigh in from patients, that it is probably safe to go this sort of non-FDA approval approach and go the off-label [approach]… I [have been going] to [***pharmaceutical company name***] to talk about [repurposing this drug candidate for our disease]. And they’re like, ‘well, we don’t really care about [***generic drug name***]’. But if you want to try [***name of drug in related class***]’, because that’s their most recent version, it has a longer patent life... But the safety profile from our perspective is not as clean and not as well defined as [***repurposed drug name***]… [N***ame of repurposed drug***] is generic, it’s off patent… So we just decided, let's not go down that path… [let’s] see what we can do on our own.” (***RDNP 21; Clinical Stage without Clinically-Significant Endpoint***)
**6. Roadblocks and challenges**	a. Lack of previous involvement in, and/or knowledge about, repurposing	"We as an organization have not been able to effectively advocate [for approval]. [The repurposing effort] just took on a life of its own. But now we're at this juncture where it's really frustrating… Not being doctors, and not being able to speak to the FDA directly… our hands are tied a little bit… [we want to understand what] developing a drug repurposing opportunity would [look like] … and then also, how to look at these opportunities in the future for other drug repurposing" (***RDNP 1; Clinically-Beneficial Off-Label Use***)<br><br>"For repurposing, [it’s] different than a brand-new drug, because so much is already known. It has already been approved for one application. So… what is going to change the [traditional] pathway from preclinical studies? Are preclinical studies even required, because those are typically in other animals, and that's already been done for an approved drug?... it's just unclear [to us]." (***RDNP 2; Clinically-Beneficial Off-Label Use***)
		“I think the biggest challenge really, what's caused the most problems here is that the fact that the patient group doesn't have a unified set of understanding about the process of scientific evidence, and because of that there's all this divisiveness about what's the right path forward." (***RDNP 20; Clinical Stage without Clinically-Significant Endpoint***)
		“So, we have candidates… being tested at [***another organization***] in neurons… [but] once we have this information… [our] small team has to try to figure out what we're going to do with it, how do we get this out there. [And], you know, a lot of what we're trying to do as an organization is [to] provide that information to them. So, for us, to have the expertise and the guidance [would] help us get things to the next level… That's kind of been our struggle within the organization. For example, we don't have our hits yet from the screen, we should have it shortly. But depending on what sort of drug candidates have been identified… without having that scientific person driving the next steps as an organization, we struggle with what do next…. That’s where I feel we need… somebody that has clinical translational drug experience.” (***RDNP 14; Abandoned***)
	b. Lack of knowledge about disease or drug-disease relationship	"We don't have good dynamic biomarkers. That’s a basic science problem that we have for the disease. And, in neurodevelopmental disorders, [endpoints] related to disability [are traditionally measured]…Are you able to improve a person's neurological development? So, for this trial that is our initial experience, [researchers] spent a lot of time coming up with a custom scale, because children with [***our disease***], typically zero out on standard things like [***endpoint 1***] and [***endpoint 2***]. So, it's really difficult to measure [using] those ways. You have this dual problem – it's very difficult to show that things are working.” (***RDNP 1; Clinically-Beneficial Off-Label Use***)

© 2025 Nijim S et al. *JAMA Network Open*.

		“The other thing I'd like to add [is that] one of the problems with [***our disease***] is that there is really no route to approval right now, other than these very difficult studies with biopsies. And I mean, we haven't seen a drug approved. So, we don't really have good endpoints in [***our disease***]. And so that’s part of the problem" (***RDNP 20; Clinical Stage without Clinically-Significant Endpoint***)
	c. Lack of physician/researcher collaboration and data sharing	"When we got to this stage, [we were] stonewalled, we [couldn’t] get information, like we were not a part of this process. It affects our lives, but we can't do anything about it. I'm making a point with everybody that we are collaborating with, to make us equal partners… we need full information…[and] we're going to take that approach with everybody that we work with." (***RDNP 1; Clinically-Beneficial Off-Label Use***)
		"I think one thing I wish I had done was get more medical professionals involved in our decision to help recruit [patients]. Afterwards, I wondered if [we] should have [pursued this project] … We don't have any MDs on our advisory board. So, there was nobody easy to ask. But I kind of wish I had maybe just reached out *ad hoc* to some people to say… do you think it's a good idea." (***RDNP 8; Abandoned***)<br><br>“The [***disease name***] field used to be extremely siloed… and has this data, [where] I need samples, and no one shares…There are 15 other [***disease name***] registries worldwide… So, you think, ‘okay, you’ll have your 30 patients in your registry’, but some of those could also be in this other registry, or you’re missing this information that can be useful for your patients…I spent six months trying to get cell lines from [***foreign country***] that we didn’t end up getting because they have such strict rules about sharing samples… So, imagine all the patient data…” (***RDNP 12; Abandoned***)
	d. Lack of pharmaceutical company support and incentive	"[***Pharmaceutical company name***] provided [***drug name***] for all of the trials. And then when it went off patent [and] they decided that they wanted to focus their efforts to other areas, so they weren't providing it anymore… I met with [***another pharmaceutical company***] who'd made this biosimilar. They're a smaller company and they are interested in helping in rare diseases. And they were very interested in [***our disease***]. And, so, they provided the drug… [and are] actively involved in meeting with the FDA and trying to get the repurposing approval." (***RDNP 2; Clinically-Beneficial Off-Label Use***)<br><br>“[***Pharmaceutical company***] didn’t listen to what we said for a long time. It took them about 18 months…and that was challenging.” (***RDNP 7; FDA-Approved***)<br><br>“We approached [***pharmaceutical company***], and they told us ‘no’. We tried for two years, actually… And then more and more physicians started dipping their toe in… I don’t know what went into that decision, but we continued to go to high-level people, and [it was still a] ‘no go’… [***Another pharmaceutical company***] was not interested… [and] didn’t give a lot of detail… [even when] we had our scientists, we had our physicians, we had patients [weighing in].” (***RDNP 11; Clinically-Beneficial Off-Label Use***)

© 2025 Nijim S et al. *JAMA Network Open*.

		“We’re dealing right now with experimental drugs where there isn’t an IIT [investigator-initiated trial] program that an investigator can apply to…companies are very, very controlling of what happens with their drug.” (***RDNP 6; Clinical Stage without Clinically-Significant Endpoint***)<br><br>“[The repurposed drug candidate] is very well tolerated. It’s easily accessible, and it’s cheap… So, there’s very low motivation to do any more work on this one [compared to] others” (***RDNP 4; Late Stage without Clinically-Significant Endpoint***)
		“We tried to get two other companies that are working now with us for other potential treatments…they discussed it internally, and they said ‘no’ for many reasons…The company decided to cut the program…they had some financial issues, so they had to cut some programs…and the program they cut was this one” (***RDNP 9; Abandoned***)<br><br>“Pharmaceutical involvement, pharmaceutical company, industry involvement, money. Once a drug is generic, they’ve already made their money… The biggest challenge has been pharmaceutical involvement” (***RDNP 25; Abandoned***)
	e. Difficulties with regulatory bodies and approval issues	"[As] the group at the NIH went through [the] clinical trial, the endpoints the FDA [placed] were so subjective, there was no way to meet them… And, so, one of the primary endpoints for the Europe trial was to measure [***endpoint 1***], the [***biochemical substance***] that's causing all of these problems. But… the FDA won't accept…the first trial in the United States [since it] did not meet [alternative] endpoints enough… It's just so subjective between one patient to another, it was difficult to prove. So now we're at a point in the United States where we're looking at all the data that was gathered in Europe, and they're meeting with a rare disease group within the CDER [Center for Drug Evaluation and Research] in the FDA. And they are working with the United States and going to the FDA meetings to get this approved…[and] getting it to a point where the FDA will agree [on alternative] endpoints" (***RDNP 2; Clinically-Beneficial Off-Label Use***)<br><br>"We thought that [***pharmaceutical company name***] was going to apply just on behalf of [our disease], but they ended up doing a batch application where they tried to push through like eight different disease designations that were in various stages of having proof points for efficacy, safety, completely different diseases… And so then basically, everything got denied. We’ve been in this extremely frustrating limbo. And, and then, now insurance companies use it as an excuse for not providing it, even though we have all of this data showing safety and efficacy." (***RDNP 1; Widespread Off-Label Use***)
		“FDA would not pass it without a full phase three clinical trial with a placebo arm. And so that’s where we’re at now… it’s so hard because especially with something like topical [***repurposed drug***], the side effects are just nothing… it’s just so frustrating to meet roadblocks from the regulatory side” (***RDNP 18; Clinical Stage without Clinically-Significant Endpoint***)
	f. Access issues and off-label concerns	"[Approval of the repurposed drug for patients] requires multiple insurance appeals…about half of people are seeing many, many months of denials. So, it's very difficult… The NHS in the UK, did a literature review… and so they actually have official guidance that people with [***our disease***] over two years of age should get [***drug name***]…Even [for] kids that manage to get insurance approval for this drug, because it’s a specialty medication, a lot of times, the copay maxes out… The first month, they have to pay $4,000.

© 2025 Nijim S et al. *JAMA Network Open*.

		[***Pharmaceutical company***] can support us with having the copay assistance support, but we [haven't had access to] that." (***RDNP 1; Clinically-Beneficial Off-Label Use***)<br><br>"We were denied by insurance companies for I think, at least three rounds ... I mean, you can always appeal and say, 'Well, no, you need to take this into consideration'… So, by the end, we had a really good package, put together that addressed all of the information… Here in North America, if I had to just buy it with no insurance coverage, it would cost me about $5,000 a month." (***RDNP 2; Clinically-Beneficial Off-Label Use***)<br><br>"When there is a commercial approval, you have to come off of managed access…people have like $6,000 copays a month and are trying to figure out how they're going to do this…A lot of people have had denials…because it's a rare disease." (***RDNP 7; FDA-Approved***)
		"Because it is off-label use… it's just a battle to get approved for coverage for it…Every insurance company negotiates their own price… we've got some people paying $27 a month, some people paying $2,500 a month" (***RDNP 24; Early Stage without Clinically-Significant Endpoint***)<br><br>"It's hard to get… Not many doctors will prescribe it. So, there's this group that is helping people find doctors who are willing to prescribe it, [and] there are some doctors who are vocally supportive. It's also very expensive. So that's part of [the problem]." (***RDNP 20; Clinical Stage without Significant Endpoint***)<br><br>"Right now, really the only way to get access to [***repurposed drug***] is through clinical trials. It was definitely a difficult endeavor, although I think it was more along the lines of getting it exported and imported… the [***foreign country***] Minister of Health got directly involved at one point to authorize the export… Getting this drug out [from ***foreign country***] was a real challenge… export was harder than import." (***RDNP 4; Late Stage without Clinically-Significant Endpoint***)
	g. Difficulties with clinical trial design, recruitment, and conduction	"Clinical trials are hard… clinical trials are messy. Finding patients with a rare disease for a trial is infinitely more difficult than what you might envision. And getting funding and getting the logistics and regulatory aspects aligned, especially if you're going internationally makes it just orders of magnitude harder. So, I think that aspect needs to be very carefully thought about in terms of the timing, the resources, the funding, and what regulatory aspects need to be considered within the organization…The trial was actually sub-powered with a sample size of 120… it took a final push with an amended protocol… in a bit of a crazy race to the finish…We ended up funding the last year of monitoring in [***foreign country***] when funds ran out… year to year, month to month, finding more money collaboratively across industry, patient organizations, [and] government." (***RDNP 16; FDA-Approved***)<br><br>"The trial itself has been extremely challenging, frankly… There was a very small trial [and was] positioned as a safety study with…10 patients. And…it was the first time that [the repurposed drug] had been administered in this population. So even though [the repurposed drug candidate] had been FDA approved…it was still the first time… and not having ever done this before [was an] issue…[and we had limited] clinical endpoint information." (***RDNP 23; Clinically-Beneficial Off-Label Use***)

© 2025 Nijim S et al. *JAMA Network Open*.

	"But, again, getting the patients was difficult... There may be five patients here, but you know, for a clinical trial, you want to have considerably more or you don't really have a good basis for making any decisions" (***RDNP 2; Clinically-Beneficial Off-Label Use***)
	"Clinical trials are really expensive [and] they're hard to do. Right now, really the only way to get access to [***repurposed drug***] is through clinical trials. It was definitely a difficult endeavor, although I think it was more along the lines of getting it exported and imported… the [***foreign country's***] Minister of Health got directly involved at one point to authorize the export… Getting this drug out [from ***foreign country***] was a real challenge… export was harder than import... The leap from N-of-1 to clinical trial is [also] enormous. And I just wish there was a better middle ground" (***RDNP 4; Late Stage without Clinically-Significant Endpoint***)
	"One of the issues that we're running up against is having siloed clinical trial approaches where you have five patients on one trial, five patients on another trial… So, one thing we're trying to be cognizant of is how are you actually going to administer it to the population in a meaningful way?" (***RDNP 12; Abandoned***)
h. Lack of patient involvement/support	"We were told by [***pharmaceutical company***] that the FDA could not take any of our information or testimony or letters or support from the [***non-profit***] or from our families, because they said it was a conflict of interest… We don't have good dynamic biomarkers… it's very difficult to measure them in those ways… It seems like [patients] really want to measure things related to disability, right? Are you able to improve a person's neurological development?" (***RDNP 1; Clinically-Beneficial Off-Label Use***)<br><br>"There is a phase two clinical trial that has a placebo arm, but…[we] did not get to weigh in on the placebo arm…that's been a bit of a roadblock." (***RDNP 7; FDA-Approved***)
	"There is a large community of clinicians and researchers in this space, and there have been for decades, which is fantastic… But because of that, there are so many players in this space, and the role that we have as a patient organization historically hasn't been as clear… [In fact] the treatment guidelines are being updated right now… [but] no patient is involved in the guidances yet… [Whether the drug will be recommended in US guidance is] a particularly emotional topic because many of the people who report a response [to the drug] are children. And some of the responses appear to be quite dramatic, they go from being very sick you know about to have a [***identifying corrective procedure***] to perfectly normal...And it's very frightening the effects that it may have on our organization's relationship with the researchers and clinicians that [***our RDNP***] has worked to develop long term relationships with. And so, for this purpose, as we talk about, drug repurposing, this could be the antithesis to what any organization would probably ever want." (***RDNP 20; Clinical Stage without Clinically-Significant Endpoint***)
	"[***University***] attempted to run a trial, but didn't actually make it to a formal clinical trial because patients weren't willing to enroll into it. And again, I think… maybe had there been more solid information behind it…maybe patients would have been more willing. I don't know. But it never rolled into an actual long-term trial. I think it only lasted a couple of months." (***RDNP 14; Abandoned***)

© 2025 Nijim S et al. *JAMA Network Open*.

		"It is difficult to keep the donors, especially the families and friends, interested in this [drug repurposing] project…at the beginning, it's like, okay, we are going to test 5,000 drugs…then we have one possible [drug]…That is a message that the patient community is not happy about" (***RDNP 8; Abandoned***)
	i. Rare disease-specific obstacles and drug-specific patent challenges	"There may be five patients here [for a rare disease], but, for a clinical trial, you want to have considerably more…The problem [also] is that no two people are the same. I mean, they are people who have multiple diseases, and [***our rare disease***] is just one of them… well, you don't want to throw out all their data, but it really is not going to be appropriate to apply that to just a [***our rare disease***] patient" (***RDNP 2; Clinically-Beneficial Off-Label Use***)<br><br>"I [had] been in pharma for about 25 years….It's hard to get a commercial partner to invest in those drugs because there's little money to be made… We know that for rare disease, your return on investment is limited. Now, if you already have a small bucket of money you will get back from that asset, and then you have to split it with five other companies… that is where I think the companies say 'okay, you know what, it's not worth our money'." *(**RDNP 5; FDA-Approved**)*<br><br>"Finding patients with a rare disease for a trial is infinitely more difficult than what you might envision… The [***name***] trial, we talked about 89 [patients], the trial was actually sub-powered with a [desired] sample size of 120" (***RDNP 16; FDA-Approved***)
		"We didn't have models at the outset…Until those things existed, very little could be done, our hands were tied… For a [***Disease Gene Name***] study, it takes a year before going from a standing start to actually running [***identifying gene***] studies because it's a slow-growing model." (***RDNP 6; Clinical Stage without Clinically-Significant Endpoint***)<br><br>"[***Name of generic drug***] is very well tolerated. It's easily accessible, and it's cheap… So there's very low motivation to do any more work on this one…No one's thinking, 'Oh, well, I need to get it indicated, so that I can get it reimbursed'. So there's very low motivation to do any more work on this one… It's not totally satisfying to me as a scientist, but it was good enough for me as a parent." (***RDNP 4; Late Stage without Clinically-Significant Endpoint***)<br><br>"I had gone to [***pharmaceutical company name***] to talk about [the repurposed drug candidate]. And they're like, 'well, we don't really care about [***generic drug name***]'. But if you want to try [***name of drug in related class***], because that's their most recent version, it has a longer patent life... The good news is, of course, is that [***name of generic drug***] is generic, it's off patent…[***The other drug***] and [***generic drug***] are essentially the same…but I would say [***the other drug***] is on the backburner because [***generic drug***] has a longer safety profile" (***RDNP 21; Clinical Stage without Clinically-Significant Endpoint***)<br><br>"Our patient population is just too small to make it economically feasible for them [pharmaceutical companies] to do it…You need 500 participants… but we don't have 500 patients in the US" (***RDNP 24; Early-Stage without Clinically-Significant Endpoint***)

© 2025 Nijim S et al. *JAMA Network Open*.

		"The FDA would not pass it without a full phase three clinical trial with a placebo arm….The FDA has required that all of our patients in the phase three be treatment naive, and so, you know, when you're a rare disease… that's been a challenge… [The drug] failed because there was no way to distinguish [effects and if the drug was still systemically circulating at [**#**] days]" (***RDNP 18; Abandoned***)
**7. Opportunities / recommendations from RDNPs**	<u>a. Patient recruitment and patient engagement at all steps of repurposing, including setting endpoints</u>	"As we know, for a rare disease, you never have enough patients in one center. So, you need to collaborate with multiple centers. That is in fact why we have all these centers that work together and say, 'I have three patients, I have five, I have seven'…We can help them with so many things. We can help them recruit the patients, we can help them get the key opinion leaders, we can help them design their clinical trials." *(**RDNP 5; FDA-Approved**)*<br><br>"We did help with enrollment, clinical trial education for our patient community…We've also helped assist in collecting data for researchers and some of their studies, since we're connected to the patient community…We connect patients with the drug company to help them, you know, design the trial…what's feasible…how far would you travel to be involved in these clinical trials?...We [tried] to help them with some of that design if we can…how about an injection? Is an infusion better? How many times can you go to the trial site?" (***RDNP 15; FDA-Approved***)<br><br>"We are big time helping with recruitment. We were delayed by COVID. We had all these problems, there was concern about recruitment, and they are [even still] going to meet their numbers early…We're supporting recruitment into that trial… And we are very confident… people are really needing this therapy…We supported compassionate use… We provided a lot of education, a lot of webinars, and at our patient conferences, we really educated the community…The patients trust us. So, we need to be part of the process. Be patient-focused, patient-driven, we should have a say in what the design is" (***RDNP 11; Clinically-Beneficial Off-Label Use***)<br><br>"We were able to collaborate with [***Pharmaceutical Company***] and tell the EMA that we will use data from [our] registry… and after four years of longitudinal data collection, we were able to obtain full EMA approval…The more organized the patient community is from the get-go, the easier it is to find patients for trials…[The EMA approval process] was another example of a collaboration between academia, our [patient] foundation-funded project, and [***Pharmaceutical Company***]… Global collaboration has really stepped up… We routinely, from a patient advocacy perspective, are on international calls, talking about what we are all going to work on together… [When submitting to the FDA], the foundation considered the citizen petition, which was the alternate route through the FDA… it was enough… bringing the patient voice to the table to say, we really need this" (***RDNP 16; FDA-Approved***)<br><br>"We have patient families who've seen really significant reduction in symptoms, so [***repurposed drug***] is definitely helping… [and we have a lot of] data showing safety and efficacy… It is being used widely off label [now]…[But, the] FDA originally rejected the [***repurposed drug***] application on safety concerns…[In addition to collecting longitudinal safety data through our registry], we're trying to set up a patient listening session with the FDA for the fall so that they better understand what living with [***disease name***] is like and the patient and family care burden…If this disease goes on unmitigated, then the consequences are dire. So, we are willing to take on these risks [from the repurposed drug and are] trying to very clearly understand, so that we can give our children opportunities that they wouldn't otherwise have" (***RDNP 1; Clinically-Beneficial Off-Label Use***)

© 2025 Nijim S et al. *JAMA Network Open*.

		"[We are trying to] find evidence-based ways to document quality of life issues, because [***repurposed drug***] seems to really help quality of life, even though it hasn't been proven to conclusively change the disease course…The most frustrating thing for patients is when [they] go off [***repurposed drug***], [they] feel worse. When [they] go back on, [they] feel better… One of our projects is the development of a quality-of-life tool that would be created in a way that the FDA would accept it as evidence. So, for both of these [drugs], that is a path—being able to document if and how these affect quality of life" (***RDNP 20; Clinically-Beneficial Off-Label Use***)
		"A big part of what we do to support a trial like that is outreach to the patient and physician community to help raise awareness about the trial, how to drive patients, help drive referrals… We got the word out to the [***disease***] patient community, and they were completely blown away because within like a week, they had their trial filled, and they'd never seen anything like this" (***RDNP 6; Clinical Stage without Clinically-Significant Endpoint***)
	<u>b. Leveraging collaboration with pharmaceutical companies and other multidisciplinary research partners</u>	"You need that internal champion that is high enough up to really champion this… and that's how this happened. So, it's really finding that internal champion in the pharmaceutical companies… We invited the head of [**pharmaceutical company**] to the [**disease name**] Conference… She came, was completely blown away by the quality of the science that was done within the [**disease**] community and said, 'I'm with you guys.' ***(RDNP 5; FDA-Approved)***<br><br>"The absolute priority has always been building a scientific network, having the grant program, investing in pilot or small grants…From day one, the focus of the mission was always research first…We were able to collaborate with [***pharmaceutical company***] and tell EMA that we will use the data from this registry…The foundation played a central role with funding as well as with organization for the [***trial 1 name***] and the [***trial 2 name***] trial" (***RDNP 16; FDA-Approved***)<br><br>"We have a pretty robust network of researchers, clinicians…that we connect with around the globe…We wrote a letter to the [***pharmaceutical company manufacturer***] and said, 'Why don't you guys do a trial?' And they responded and decided to do a clinical trial…We [also initially] connected with organizations like the National Organization for Rare Disorders and Global Genes and [***identifying society***] and the [***identifying American research society***]…[when] we didn't really know where to start…we didn't want to reinvent the wheel so we reached out to other groups that were doing this [and learned] what their best practices were" (***RDNP 15; FDA-Approved***)<br><br>"That meeting that happens every year is very key, all the important players around the world are there and can give their input…that makes it easier in some ways to make a decision and move forward…It's easier when the nonprofit can bring those researchers together…And I actually went maybe six years ago to the UK because we were contacted by [***pharmaceutical company***] who has made another drug, now that it's off patent, they have their own version, which is a little bit different. It doesn't require refrigeration [like the other one]… And they are working with the United States and going to the FDA meetings to get this approved under repurposing" (***RDNP 2; Clinically-Beneficial Off-Label Use***)<br><br>"I think the other aspect [compared to challenges faced by other projects and organizations]… is we had a PFDD [patient-focused drug development]. And [we], actually, really have found ways to enhance communication and interest with pharmaceutical companies… so, with the PFDD with all of the work that we've been doing with pharma [companies], they're starting to come to us now. And we have

		players or partners in the industry who will advocate on our behalf when they hear a new drug company is thinking of pursuing [***our disease***]" (***RDNP 20; Not Discussing Specific Project; Previous Clinically-Beneficial Off-Label Use***)
		"The group in [***foreign country***] had a pre-existing relationship with [***lead physician in foreign country***], and was able to convince them to provide the drug and partial support for the trial…The only way to get that done is to be able to hand it to them on a plate. And just do it for them… We said, okay, it doesn't make sense to fund all three of these [***drug class inhibitors***] because they're all very similar…Let's see if we can get these groups to work together." (***RDNP 6; Clinical Stage without Clinically-Significant Endpoint***)<br><br>"[***RDNP website name***] was helpful in bringing researchers together… there was really no funding, it was more of a networking thing, connecting researchers together once there was a hit… As part of one of my broader research collaborations… there's a [government]-funded rare disease clinical research network associated with [our RDNP]. And that rare disease clinical research is funding clinical trials now." (***RDNP 4; Late-Stage without Clinically-Significant Endpoint***)
	c. Establishing forms of systematic data collection and crowdsourcing research and research ideas	"That's why we have a patient registry, both for recruitment, but also for solving a real heavy problem that we have, which are clinical outcomes… [Using this], we should be able to demonstrate that a lot of events that the FDA might be concerned about are happening regardless of treatment, and to help tease it away from the trial." (***RDNP 1; Clinically-Beneficial Off-Label Use***)<br><br>"We have a natural history study that we've been conducting for the last five years, so we [have been] collecting data for that [after we were established]… We looked around and tried to figure out what the best platform was, and we ended up going with the NORD [National Organization of Rare Diseases, non-profit] IAMRARE platform [after] receiving a grant to join that platform…We've [used our platform to] assist in collecting data for researchers and some of their studies, since we're connected to the patient community…[and connect researchers with] patient reported data… but not a lot on the funding side" (***RDNP 15; FDA-Approved***)<br><br>"The NIH history trial…that is ongoing, that's been going for many years, almost 20 years…and, alongside that, there was a clinical trial at the NIH as well, where they invited people who were participating in the history trial to join that trial if they were interested… And that [off-label use and clinical outcomes] is one of the things that [the larger international umbrella organization focused on their rare disease] tracks. And all of it is accessible by all of the societies" (***RDNP 2; Clinically-Beneficial Off-Label Use***)
		"We have an internal anecdotal response list, basically… when we hear about a response [regarding a possible drug or off-label use], we [keep track of it]…for hypothesis-generating purposes" (***RDNP 6; Clinical Stage without Clinically-Significant Endpoint***)<br><br>"We started a large collaborative consortium called [***name***] in [***year***]… I call it the 'football model'…First, patients contact us [with a research question]… [And] the whole idea is we bring together experts from all over the world [to address the question]. So, we really needed to have people with very different skill sets that are part of the [***name***] team. And, the ultimate client of what we're doing has to be part of consortium, so we have patients in the consortium. We say, 'Okay, you guys, we put $3 million on the table', [and all researchers] submit something to solve this conundrum... So, [everyone] came together: people that were doing cell models, animal models, transcriptomic [and] proteomic studies… [***Repurposed drug candidate***] was incredibly powerful [in cell and animal models]…

© 2025 Nijim S et al. *JAMA Network Open*.

		[but the transcriptomic and proteomic studies were also needed] to actually figure out that this molecule was hitting mechanisms that were super relevant for [***disease***].” (***RDNP 5; Clinical Stage without Clinically-Significant Endpoint***)<br><br>“We create [research] programs… seeing what expertise [researchers] have and saying I want you to work on this part of the project… and you’re all going to work together and share resources…What we’re working on now is trying to connect all those registries to maybe a global unique identifier or some sort of way to standardize all the data collection” (***RDNP 12; Early-Stage without Clinically-Significant Endpoint***)<br><br>“We’ve started asking people to report what substances they’re taking, and we’re going to track and measure their progress…[***Name***] over in the UK started taking [repurposed drug] with antioxidants… Within six months, he showed a visible reduction in [***pathological substance***] on his [***identifying imaging***]…We’ve applied for a natural history study grant from the FDA…[and] we’re trying to get a clue as to how the disease progresses… We’ll collect blood samples and [***identifying specimen***] samples to try and look for biomarkers” (***RDNP 24; Early-Stage without Clinically-Significant Endpoint***)
	d. Exploring alternative models for clinical trial execution	“I think that the future will probably see the [***RDNP name***] running a platform trial… it kind of gives us the power where, otherwise, we’re getting scraps of information.” (***RDNP 1; Clinically-Beneficial Off-Label Use***)<br><br>“[For the clinical trial], we outsourced. It’s a phase two, three [trial]… We have outsourced a CRO [contract research organization]… We have 12 of our centers, we have a central IRB with [***hospital name***]” (***RDNP 11; Clinically-Beneficial Off-Label Use***)<br><br>“After [***Pharmaceutical company manufacturer***] made the decision against [submitting materials to the FDA for a label change], you can [conduct a trial] under what’s called an IND exemption…you don’t have to take the 1700 additional steps that are really expensive and time-consuming, that just make your trial way, way more expensive (***RDNP 3; Clinically-Beneficial Off-Label Use***)
		“We built together with [***hospital system name***] and [***pharmaceutical company name***], honestly, a platform trial called [***name***]. And that drug is now in the platform trial, which are six sites across the US, and they’re running that drug through this multi-[disease], multi-site trial” *(**RDNP 5; Clinical Stage without Clinically-Significant Endpoint**)*

© 2025 Nijim S et al. *JAMA Network Open*.

	<u>e. Facilitating access when alternative endpoints set</u>	"By the end, we had a really good package put together that addressed all of the information. And, so, [after] sharing that with other physicians, there's been no one that's been denied this package to use it off-label…We push [for coverage using] the [***mechanism***], because…if you can stop that, then none of those [cost-intensive] things will happen [down-stream]. And so that [cost-efficacy] case is made" (***RDNP 2; Clinically-Beneficial Off-Label Use***)<br><br>"And they're still using it off label… In fact, a lot of kids in the United States… It requires multiple insurance appeals, but we have resources on our website, where we lay it all out for people" (***RDNP 11; Clinically-Beneficial Off-Label Use***)<br><br>"Outside, some other countries in Europe have had some success [with insurance approvals for the repurposed drug], [but it] typically requires a very knowledgeable and proactive doctor. So, we have some resources on our website, where we lay it all out for people: the benefits, these are the studies, we have a template insurance appeal letter written for them. That's sort of the approach that we've taken. There are a lot of people doing it off label…" (***RDNP 1; Clinically-Beneficial Off-Label Use***)

© 2025 Nijim S et al. *JAMA Network Open*.
