# Supplementary material for: Rare Disease Drug Repurposing
Source: JAMA Netw Open. 2025 May 5;8(5):e258330. doi: 10.1001/jamanetworkopen.2025.8330 (PMC12053540; doi:10.1001/jamanetworkopen.2025.8330)
Supplement: Supplement 3. — Data Sharing Statement [file jamanetwopen-e258330-s003.pdf]

Data Sharing Statement

Nijim. Rare Disease Drug Repurposing. *JAMA Netw Open*. Published May 05, 2025.
doi:10.1001/jamanetworkopen.2025.8330

Data
Data available: Yes
Data types: Deidentified participant data
How to access data: Deidentified participant data in the form of quotes provided during semi-structured interviews are available in eTable 3. Publicly-available characteristics of nonprofit organizations comprising the study population are also available in eTable 2. The full deidentified survey response dataset and model outputs can be made available upon request to those requesting the data for analysis purposes.
When available: With publication

Supporting Documents
Document types: None

Additional Information
Who can access the data: The aforementioned data is made available in the Supplement section to all viewers. Data sharing will be considered for all reasonable analytic requests.
Types of analyses: The data can be available for analytic purposes.
Mechanisms of data availability: The deidentified participant data noted above will be made available through the Online Supplement section of the manuscript. The full deidentified survey response dataset and model outputs can be made available for the purposes outlined above upon request to the corresponding authors.
